# Veterans Health Administration (VA) vs. Non-VA Healthcare Quality: A Systematic Review

**DOI:** 10.1007/s11606-023-08207-2

**Published:** 2023-04-19

**Authors:** Eric A. Apaydin, Neil M. Paige, Meron M. Begashaw, Jody Larkin, Isomi M. Miake-Lye, Paul G. Shekelle

**Affiliations:** 1 Evidence Synthesis Program, VA Greater Los Angeles Healthcare System, Los Angeles, CA USA; 2 RAND Corporation, Santa Monica, CA USA; 3 Department of Medicine, David Geffen School of Medicine, University of California, Los Angeles, CA USA; 4 RAND Corporation, Pittsburgh, PA USA; 5 Department of Health Policy and Management, Fielding School of Public Health, University of California, Los Angeles, CA USA

**Keywords:** quality, VA, inpatient, outpatient, community care, safety, cost, patient experience, access

## Abstract

**Background:**

The Veterans Health Administration (VA) serves Veterans in the nation’s largest integrated healthcare system. VA seeks to provide high quality of healthcare to Veterans, but due to the VA Choice and MISSION Acts, VA increasingly pays for care outside of its system in the community. This systematic review compares care provided in VA and non-VA settings, and includes published studies from 2015 to 2023, updating 2 prior systematic reviews on this topic.

**Methods:**

We searched PubMed, Web of Science, and PsychINFO from 2015 to 2023 for published literature comparing VA and non-VA care, including VA-paid community care. Records were included at the abstract or full-text level if they compared VA medical care with care provided in other healthcare systems, and included clinical quality, safety, access, patient experience, efficiency (cost), or equity outcomes. Data from included studies was abstracted by two independent reviewers, with disagreements resolved by consensus. Results were synthesized narratively and via graphical evidence maps.

**Results:**

Thirty-seven studies were included after screening 2415 titles. Twelve studies compared VA and VA-paid community care. Most studies assessed clinical quality and safety, and studies of access were second most common. Only six studies assessed patient experience and six assessed cost or efficiency. Clinical quality and safety of VA care was better than or equal to non-VA care in most studies. Patient experience in VA care was better than or equal to experience in non-VA care in all studies, but access and cost/efficiency outcomes were mixed.

**Discussion:**

VA care is consistently as good as or better than non-VA care in terms of clinical quality and safety. Access, cost/efficiency, and patient experience between the two systems are not well studied. Further research is needed on these outcomes and on services widely used by Veterans in VA-paid community care, like physical medicine and rehabilitation.

**Supplementary Information:**

The online version contains supplementary material available at 10.1007/s11606-023-08207-2.

## INTRODUCTION

The Veterans Health Administration (VA) is the nation’s largest integrated healthcare system, providing care for millions of U.S. military Veterans. Providing high quality of healthcare is a commitment VA makes to Veterans. Comparisons of VA-delivered care to care delivered in non-VA settings is central to assessing the quality of VA healthcare. Prior reviews conducted in 2011 and 2016 comparing outcomes between VA and non-VA care included data through 2014, and found that VA care performed similarly to or better than non-VA care in most, but not all, aspects of healthcare quality.^1–3^

Since that time concerns about access to care led to the Veteran Access, Choice, and Accountability (“Choice”) Act of 2014, which allowed Veterans to seek medical care in the community if the VA was unable to schedule a visit within 30 days or if the Veteran lived greater than 40 miles from their closest VA. This program also required independent performance assessments of VA’s healthcare services related to access and available expertise.^4^ Choice Act funding ended in 2017 and was followed by the VA Maintaining Internal Systems and Strengthening Integrated Outside Networks (MISSION) Act of 2018 that further addressed concerns regarding Veteran access to care by expanding eligibility for VA-reimbursed community care (CC) options.^5^ These acts greatly expanded the potential for care delivered to Veterans and paid for by VA to be from community providers, raising additional questions about comparisons of the quality of healthcare. Unlike prior studies and reviews of prior studies where subjects sorted into VA or non-VA care due to eligibility or preference, there is now the situation where VA is enabling Veterans eligible for VA care to get care outside of VA – community care – at VA’s expense.

To address these gaps and update the understanding of Veteran care outcomes, we conducted a systematic review to compare clinical quality and safety, access, patient experience and cost between VA and non-VA medical care, with a particular focus on comparisons of the quality of healthcare between VA care and VA-paid community care.

## METHODS

This review is part of a larger review commissioned by the Veterans Health Administration detailing differences between healthcare quality of VA and non-VA medical and surgical care.^6^ The protocol for this larger review was preregistered on PROSPERO (http://www.crd.york.ac.uk/PROSPERO/; registration number [CRD42022314154]).

### Data Sources and Searches

We conducted broad searches using terms relating to “Veterans health” and “community health services” or “private sector.” To identify articles relevant to the key questions, a research librarian searched PubMed, Web of Science, and PsychINFO (January 2015 to March 2023). The start date was chosen to match the end date of the most recent review by O’Hanlon, et al.^2^ Additional citations were identified from hand-searching reference lists and consultation with content experts. We limited the search to published and indexed articles involving human subjects available in the English language. See Supplementary Material 1 for complete search strategy.

### Study Selection, Data Abstraction, and Study Quality Assessment

Team members working independently screened the titles of retrieved citations, and titles flagged for potential inclusion were screened by a second team member. All disagreements were reconciled through group discussion. Full-text review was conducted in duplicate by independent team members with any disagreements resolved through discussion. All data abstraction were first completed by one reviewer and then checked by another; disagreements were resolved by consensus or discussion with an additional reviewer. Study selection criteria and data abstraction items are detailed in Supplementary Material 2. The risk of bias for these studies was judged by the representativeness of their samples being assessed and whether the measures of performance in the studies were valid and were applied equally across both study groups. Study quality assessment criteria and categorization are detailed in Supplementary Material 2 and 3.

### Synthesis

Studies were summarized by a narrative synthesis. Studies were first classified into one or more Institute of Medicine healthcare quality domains:^7^ clinical quality and safety, access, patient experience and cost/efficiency. Within domains studies were grouped by clinical condition (cardiovascular, etc.). Studies failing one or more of the above quality criteria were not included in the synthesis (See Supplementary Material 4).

## RESULTS

### Search Results and Synthesis Overview

**Figure 1:**
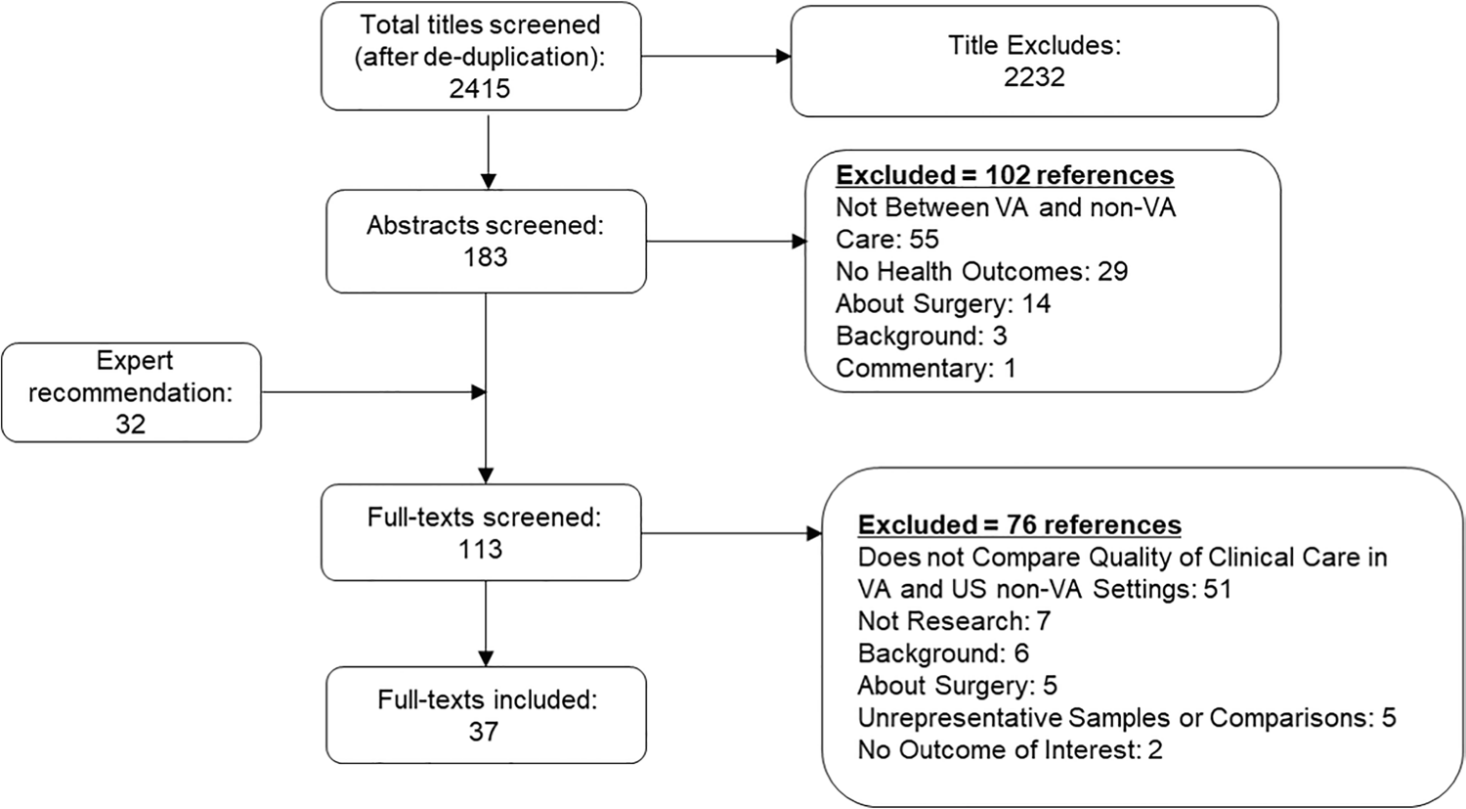
Literature flowchart.

**Figure 2:**
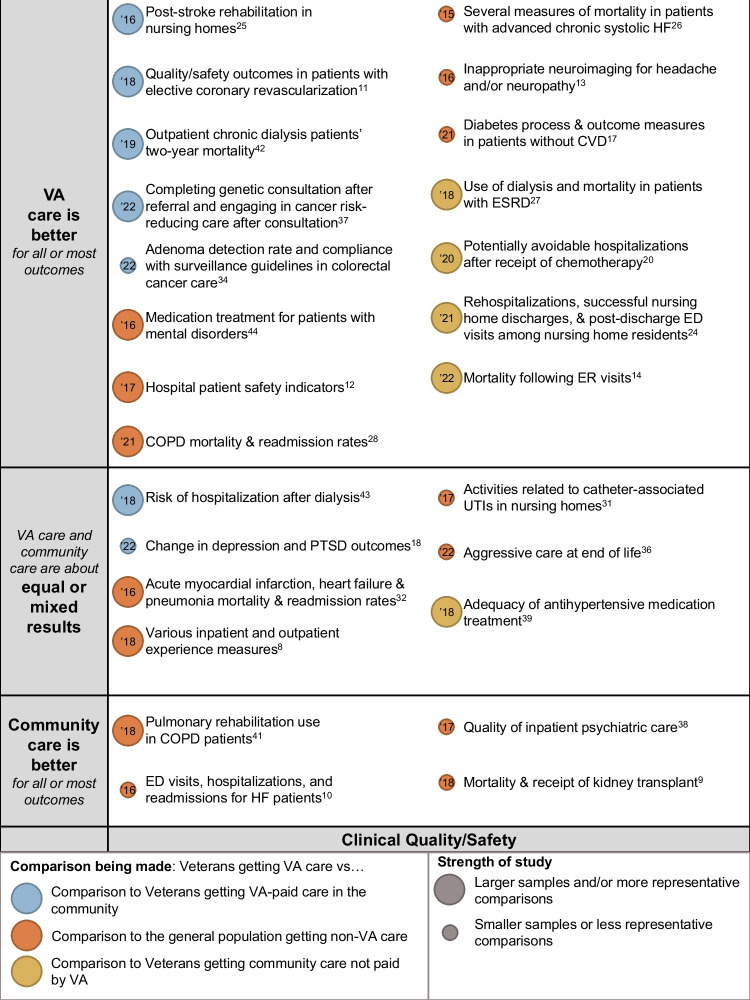
Evidence map of VA vs. non-VA clinical quality/safety. Notes: Studies are listed on the vertical axis by their qualitative results (VA care is better than community care, VA care and community care are about equal, or results are mixed, and community care is better than VA care). Study quality is depicted by bubble size, with larger bubbles being studies of better quality and representativeness than studies depicted by smaller circles. The color of the bubble indicates the type of comparison: blue for studies comparing Veterans getting care from VA to Veterans getting VA-paid care in the community; orange for studies comparing Veterans getting care from VA and non-Veterans, or a general population, getting care in the community; and, yellow for studies comparing Veterans getting care from VA to Veterans getting care in the community not paid for by VA. Beside each circle is a brief annotation of the study topic, and inside the bubble is the year of publication (’18 = 2018,’19 = 2019, etc.).

**Figure 3:**
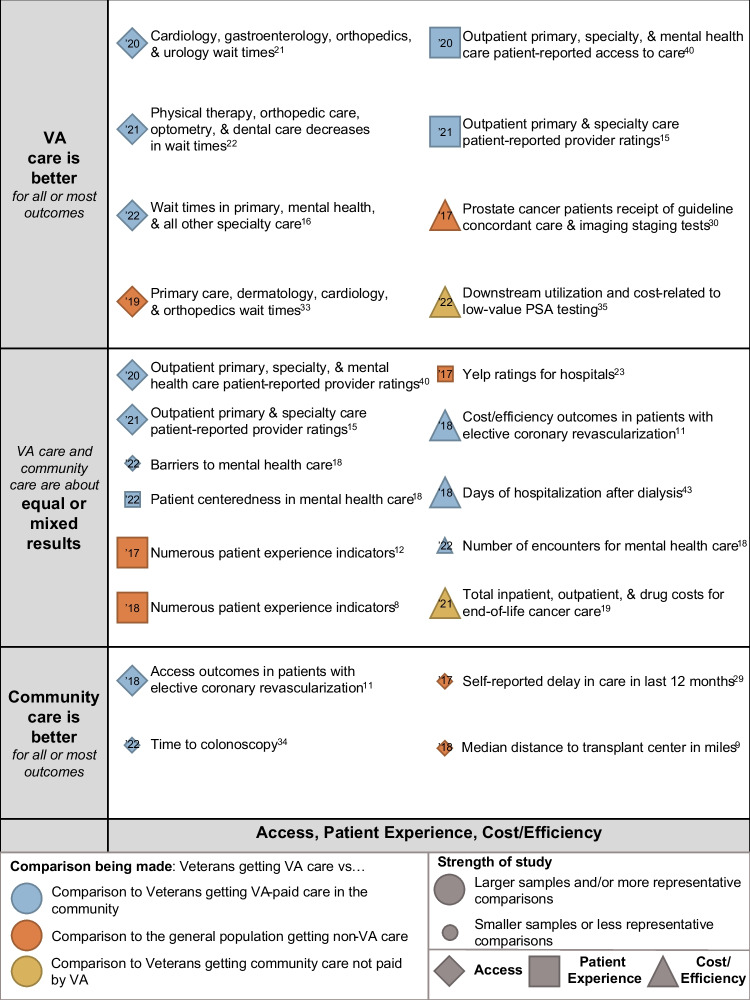
Evidence map of VA vs. non-VA access, patient experience, and cost/efficiency. Notes: Studies are listed by domains of care of the outcomes they report by shape: diamonds for access; squares for patient experience; and triangles for cost/efficiency. Studies are also listed on the vertical axis by their qualitative results (VA care is better than community care, VA care and community care are about equal, or results are mixed, and community care is better than VA care). Study quality is depicted by bubble size, with larger bubbles being studies of better quality and representativeness than studies depicted by smaller circles. The color of the bubble indicates the type of comparison: blue for studies comparing Veterans getting care from VA to Veterans getting VA-paid care in the community; orange for studies comparing Veterans getting care from VA and non-Veterans, or a general population, getting care in the community; and, yellow for studies comparing Veterans getting care from VA to Veterans getting care in the community not paid for by VA. Beside each circle is a brief annotation of the study topic, and inside the bubble is the year of publication (’18 = 2018,’19 = 2019, etc.).

We identified 37 studies^8–44^ that met inclusion criteria, after successively screening 2415 titles after deduplication from 2448 titles, 183 abstracts, and 113 full-text articles (Fig. 1; full list of excluded studies available in Supplementary Material 4; evidence table in Supplementary Material 5). Our overall results are presented in the bubble plot/evidence maps in Figures 2 and 3. Twelve of these studies compared VA and VA-paid community care.

### Risk of Bias/Study Quality

Twenty-five included studies^8,11,12,14–16,19–22,24,25,27,28,30,32,33,35,37,39–44^ met all our risk of bias criteria. These good quality studies were given more weight in our narrative synthesis than studies that did not meet one or more criterion. Twelve studies^9,10,13,17,18,23,26,29,31,34,36,38^ met some of our criteria at the middle study quality level, and were categorized as fair study quality (for reasons, see Supplementary Material 3). We included all these studies but gave them less weight when reaching our conclusions. Seven studies were rated at the lowest study quality level for one or more of our criteria, and were excluded from analyses entirely. See Supplementary Material 4 for the excluded studies and Supplementary Material 6 for the risk of bias table.

### Clinical Quality and Safety

Twenty-six studies^8–14,17,18,20,24–28,30–32,34,36–39,41–44^ reported clinical quality outcomes. These outcomes were reported in five studies of cardiovascular disease,^10,11,26,32,39^ three studies of nursing home care,^24,25,31^ four studies of dialysis and end stage renal disease,^9,27,42,43^ three studies of mental health conditions,^18,38,44^ two studies of hospital safety indicators and outpatient clinical quality of care,^8,12^ two studies of cancer,^34,36^ two studies of chronic obstructive pulmonary disease,^28,41^ and five studies of miscellaneous conditions.^14,20,37,13,17^ Sixteen studies^8,11,12,14,20,24,25,27,28,32,37,39,41–44^ were of study good quality, and ten^9,10,13,17,18,26,31,34,36,38^ were of fair study quality. VA care was better in 15 of these studies,^11–14,17,20,24–28,34,37,42,44^ including 5 studies^11,25,34,37,42^ comparing VA and VA-paid community care. Clinical quality or safety was equal or mixed between VA and non-VA care in 7 studies,^8,18,31,32,36,39,43^ and worse in VA in 4 studies.^9,10,38,41^

#### Cardiovascular Disease Outcomes

We identified five studies, three^11,32,39^ of good study quality and two^10,26^ of fair study quality, that compared cardiovascular outcomes. In two studies VA care was better,^11,26^ in two studies VA and non-VA care was equal or outcomes were mixed,^32,39^ and in one study VA care was worse.^10^ Mortality was better for cardiovascular revascularization patients in the only study (VA: n = 15,340; non-VA: n = 3715) comparing VA and VA-paid community care (CC; VA: 0.65% vs. non-VA: 1.54%, p < 0.001).^11^ Mortality and readmission rates were similar for patients with advanced chronic systolic heart failure in VA and non-VA hospitals, despite VA patients being older and sicker, in an analysis of data from the Beta-blocker Evaluation of Survival Trial (BEST; VA: n = 898; non-VA: n = 1216).^26^ In two other studies, VA care was similar to non-VA care. An analysis (VA: n = 7929–26,231; non-VA: n = 124,220–269,856) of patients with for acute myocardial infarction (AMI), heart failure (HF), and pneumonia between VA and non-VA hospitals showed that mortality and readmission rates did not consistently favor either system.^32^ Similar mixed results were reported in a study (VA: n = 35,647; non-VA: n = 9922) of the adequacy of antihypertensive medication supply for dementia patients receiving care by VA or Medicare.^39^ One study showed that VA care was worse than non-VA care.^10^ In this study (VA: n = 2242; non-VA: n = 8825), heart failure patients in non-VA care were more likely to experience fewer emergency department visits (odds ratio [OR] 0.62, 95% confidence interval [CI] 0.60–0.64), hospitalizations (OR 0.98, 95% CI 0.95–1.02), and readmissions (OR 0.87, 95% CI 0.83–0.90), compared to patients receiving VA care.

#### Nursing Home Care Outcomes

Three studies, two^24,25^ of good and one^31^ of fair study quality, were included that compared a national sample of clinical quality and safety outcomes in VA Community Living Centers (CLC) versus nursing homes (NH) in the community. Clinical quality was better in VA in two studies,^24,25^ and equal to non-VA care in another study.^31^ In one study (VA: n = 23,839; non-VA: n = 1,674,578) using risk-adjusted analyses, patients had lower rates of emergency department visits (VA: 8.27% vs. non-VA: 11.85%, p < 0.001) and higher rates of successful discharges (VA: 67.74% vs. non-VA: 57.04%, p < 0.001) in VA CLCs versus community NHs, but rehospitalization rates were worse in VA (VA: 22.5% vs. non-VA: 21.1%, p < 0.001).^24^ Results were better in VA in a study (VA: n = 12,660; non-VA: n = 5612) of post-stroke patients in nursing homes: VA CLC patients required and received less rehabilitation therapy, and received more restorative nursing care compared to VA-contracted community NHs.^25^ Activities to prevent catheter-associated urinary tract infection (CAUTI) were also neither consistently better nor worse in VA in a third study (VA: n = 47 facilities; non-VA: n = 306 facilities) comparing VA CLCs and community NHs.^31^

#### Dialysis and end-Stage Renal Disease Outcomes

We also included four studies, three^27,42,43^ of good study quality and one^9^ of fair study quality, which compared clinical quality outcomes for Veterans receiving care for end-stage renal disease (ESRD) or for dialysis through the VA or outside the VA. VA clinical quality was better in two studies,^27,42^ equal in one study,^43^ and worse in one study.^9^ In two studies, 2-year mortality was lower in VA care versus non-VA care in the community for ESRD (VA: n = 1100; non-VA: n = 18,215; VA: 32.4% [95% CI 29.2% to 35.4] vs. Medicare: 36.7% [95% CI 36.0% to 37.4%] vs. VA Purchased Care: 36.0% [95% CI 34.0% to 38.0%])^42^ and pre-ESRD patients (VA: n = 2966; non-VA: n = 2966; VA: 44% vs. Medicare: 53%; adjusted risk difference: 6% [95% CI 4% to 8%]).^27^ Another study (VA: n = 1101; non-VA: n = 3805 [VA Purchased Care], 18,267 [Medicare]) found that risk of hospitalization after dialysis was similar in VA and non-VA settings (p < 0.0001, but authors noted that the differences found were so small as to not be clinically meaningful).^43^ In a final study (VA: n = 3663; non-VA: n = 297,794), VA performed worse, as kidney transplantation was less likely for VA patients compared to patients with private insurance (hazard ratio [HR] 0.72, 95% CI 0.68–0.76) or Medicare (HR 0.85, 95% CI 0.81–0.90) receiving non-VA care.^9^

#### Mental Health Conditions

We included three national studies, one of good^44^ and two of fair^18,38^ study quality, that assessed clinical quality and safety outcomes for Veterans and non-Veterans with mental health conditions. VA clinical quality was better in one study,^44^ mixed in one study,^18^ and worse in the last study.^38^ The good quality study (VA: n = 836,519; non-VA: n = 545,484)^44^ found better clinical quality of medication treatment among VA-treated patients versus non-VA-treated patients. One fair quality study (VA: n = 303; non-VA: n = 242) found lower depression symptoms and equivalent posttraumatic stress disorder symptoms among Veterans receiving in-person, VA-paid community care, compared to those who received VA tele-mental healthcare.^18^ The other fair quality study (VA: n = 105 facilities; non-VA: n = 141 facilities) found worse clinical quality of inpatient psychiatric care in VA hospitals compared to non-VA hospitals.^38^

#### Hospital Patient Safety Indicators and Outpatient Clinical Quality of Care

We identified two good quality studies that compared a number of clinical quality indicators in inpatient (e.g., death among surgical patients with treatable conditions, pressure ulcers, iatrogenic pneumothorax, etc.) and outpatient (e.g., breast and colorectal cancer screening, blood pressure control among patients with hypertension, eye examination among diabetics, etc.) care.^8,12^ Both studies assessed national samples for both VA and non-VA care, including more than 100 VA facilities and hundreds or thousands of non-VA facilities. In the first study (VA: n = 129 facilities; non-VA: n = 4010 facilities), clinical quality of inpatient care in VA was better than non-VA care for most measures.^12^ In the second study (VA: n = 135 facilities; non-VA: n = 402 facilities), VA clinical quality of inpatient and outpatient care was better for many measures, however, VA had higher 30-day risk-standardized readmission rates than non-VA care.^8^

#### Cancer Outcomes

Two studies^34,36^ of cancer care, both of fair quality, also met our inclusion criteria. In the first study^34^ (VA: n = 235; non-VA: n = 235) of colorectal cancer care, the adenoma detection rate (OR 0.39, 95% CI 0.25 to 0.63) and compliance with surveillance guidelines (OR 0.21, 95% CI 0.09 to 0.45) was worse in non-VA compared to VA. In the second study^36^ (VA: n = 18,054; non-VA: n = 13,277) of nonsmall long cancer, aggressive care at end of life in some measures declined more significantly in VA (p < 0.001) compared to non-VA from 2006 to 2012. For other measures, there was no difference between systems.

#### Chronic Obstructive Pulmonary Disease (COPD) Outcomes

Clinical quality outcomes for COPD patients were described in two good quality studies. VA clinical quality was better in the first study^28^ and worse in the second study.^41^ Thirty-day readmission rates (VA: 15.3% [95% CI 11.8% to 20.2%] vs. non-VA: 19.5% [95% CI 15.8% to 26.1%]) and mortality rates (VA: 6% [95% CI 3.2% to 10.5%] vs. non-VA 8.5% [95% CI 4.9% to 14.3%]) for COPD patients were significantly lower in VA versus non-VA hospitals in one study (VA: n = 126; non-VA: n = 3523).^28^ In another study (VA: n = 32,856; non-VA: n = 158,137), VA performed worse, with lower pulmonary rehabilitation rates among VA patients hospitalized for COPD (1.5%) than Medicare beneficiaries with the same condition (2%; p-value not reported.^41^

#### Miscellaneous Conditions

We identified for 5 studies, three^14,20,37^ of good study quality and two^13,17^ of fair study quality, that reported clinical quality and safety outcomes in miscellaneous conditions. VA clinical quality and safety was better in all studies. In the first study (VA: n = 231,611; non-VA: n = 1,238,546), patients transported by ambulance to an emergency department had lower 30-day mortality per 100 patients in VA hospitals compared to non-VA hospitals (VA: 9.32 [95% 9.15 to 9.50] vs non-VA: 11.67 [95% CI 11.58 to 11.76).^14^ In the second study (VA: n = 9522; non-VA: n = 17,921), hospitalizations that could have been avoided following chemotherapy were higher among Veterans receiving care through Medicare than through VA (OR 1.58, 95% CI 1.41–1.78).^20^ VA care was also better than non-VA care in a study (VA: n = 35,647; non-VA: n = 9922) analyzing measures of diabetes control^17^ and in a study (VA: n = 256,608; non-VA: n = 2005) of possibly inappropriate neuroimaging studies in patients presenting with headache or neuropathy.^13^ In the last study (VA: n = 6775; non-VA: n = 3423),^37^ Veterans completed genetic consultations they were referred for less often in VA-paid community care (OR 0.43, 95% CI 0.28 to 0.65), compared to VA care. Patients who had VA-paid community care genetic consultations were also less likely to receive follow up cancer surveillance and risk-reducing procedures (OR 0.64, 95% CI 0.52 to 0.78) than patients in VA care.

### Access

Eleven studies^9,11,15,16,18,21,22,29,33,34,40^ reported outcomes related to access. Five of these studies described wait times,^16,21,22,33,34^ three listed different patient-reported access outcomes,^15,18,40^ two reported distance to VA or non-VA facilities,^8,10^ and one noted self-reported delays in care.^28^ Seven^11,15,16,21,22,33,40^ of these studies were of good study quality, while four^9,18,29,34^ were of fair study quality. In four of these studies,^16,21,22,33^ three of which compared VA and VA-paid community care,^16,21,22^ VA access was better. In three other studies,^15,18,40^ access was equal in VA and non-VA care, and in four studies,^9,11,29,34^ access in VA was worse.

#### Wait Times

Four good quality studies^16,21,22,33^ and one fair quality study^34^ evaluated wait times in various primary and specialty care settings. VA wait times were better or improved more in four studies,^16,21,22,33^ and were worse in one study.^34^ In the first study (VA: n = 15–30 metropolitan areas; non-VA: n = 15–30 metropolitan areas),^33^ wait times for outpatient primary care, dermatology, cardiology, and orthopedics visits in VA decreased from a mean of 22.5 days (SD 7.3 days) in 2014 to 17.6 days (SD 4.9 days; p = 0.046) in 2017, to be shorter than private sector wait times in that year (p < 0.001). Mean private sector wait times of 18.7 days (standard deviation [SD] 7.9 days) in 2014, which were nonsignificantly shorter than VA wait times during that year, increased by a nonsignificant amount (4.8 days [SD 5.2 days]; p-value not reported) from 2014 to 2017. In the second study (VA: n = 420,590–487,014; non-VA: n = 76,706–150,429), wait times between fiscal year (FY) 2015 and FY 2018 for outpatient physical therapy, optometry, orthopedics, and dental care declined more significantly for VA care compared to VA-paid community care for urban and rural Veterans.^22^ Cardiology wait times declined at the same rate in both systems. In the third study (VA: n = 2,504,355; non-VA: n = 533,609), overall wait times in outpatient cardiology, gastroenterology, orthopedics, and urology between 2018 and 2019 were lower in VA care compared to VA-paid Choice care (VA 41.1, SD 15.9 days vs non-VA 49.0, SD 15.5 days; p-value not reported).^21^ In the fourth study (VA: n = 4,016,156; non-VA: n = 3,042,060),^16^ wait times from 2018 to 2021 were shorter in adjusted analyses were lower in VA care compared to VA-paid community care in 15 of 18 VISNs for primary care, in 16 of 18 VISNs for mental health care, and in 17 of 18 VISNs for all other specialty care (p-values not reported). In the last study (VA: n = 235; non-VA: n = 235),^34^ time to colonoscopy was significantly longer in VA (83.8 days, 95% CI 45.2 to 122.4), compared to VA-paid community care (58.4 days, 95% CI 24.7 to 92.1 days; p < 0.0001).

#### Patient-Reported Access Outcomes

Patient-reported access to care was mixed in two good quality studies,^15,40^ and one fair quality study.^18^ VA and VA-paid CC patients, in one study (VA: n = 29,095–432,218; non-VA: n = 29,095–432,218), rated access to specialty care as better in the second quarter of 2016, but no different for primary and mental health care, and ratings for all three care types did not differ over time between VA and VA-paid CC by the fourth quarter of 2017.^40^ In a second analysis (VA: n = 1,019,732; non-VA: n = 63,638), rural Veterans reported better satisfaction with access to care in FY16 and FY19 in VA compared to VA-paid CC, but satisfaction with access to specialty care was similar between groups. Urban Veterans reported worse satisfaction with access to VA care in both years.^15^ In the last analysis (VA: n = 303; non-VA: n = 242), VA patients reported more access-related barriers to mental health care compared to patients receiving VA-paid community care (p < 0.001).^18^

#### Other Access Outcomes

A good quality study,^11^ and two fair quality studies^9,29^ reported other access outcomes. VA access was worse in all three studies.^9,11,29^ Authors of the first study (VA: n = 15,340; non-VA: n = 3715)^11^ found that VA patients traveled farther than CC patients for both percutaneous coronary intervention (PCI; VA: 90.8 miles vs. non-VA: 60.1 miles; p < 0.001) and coronary artery bypass graft (CABG; VA: 123.2 miles vs. non-VA: 81.5 miles; p = 0.02). Patients also incurred higher travel costs in VA for both PCI (VA: $238 vs. non-VA: $198; p = 0.004) and CABG (VA: $958 vs. non-VA: $630; p < 0.001). The second (VA: n = 3663; non-VA: n = 297,794) and third (VA: n = 203; non-VA: n = 10,719) studies, VA patients lived farther away from kidney transplant centers than patients using Medicare or private insurance,^9^ and were more likely to report delays in seeking care than patients using Medicare, Medicaid, or commercial insurance.^29^

### Patient Experience

Six studies^8,12,15,18,23,40^ reported patient experience outcomes. Two studies^15,40^ described ratings of providers, three studies^8,12,18^ reported various patient experience measures, and another^23^ reported Yelp ratings of hospitals. Four^8,12,15,40^ of these studies were good quality, and two^18,23^ were of fair quality. Patient experience between VA and non-VA care was equal or mixed in four studies.^8,12,18,23^ VA patient experience was better in two studies,^15,40^ both of which compared VA to VA-paid community care.

The first study (VA: n = 29,095–432,218; non-VA: n = 29,095–432,218) reported that provider ratings for primary, specialty, and mental health care were higher for patients in VA care compared to those in VA-paid CC care.^40^ These ratings did not change over time. Rural and urban Veterans in another study (VA: n = 1,019,732; non-VA: n = 63,638) also rated providers in primary and specialty care in VA more highly than providers in VA-paid CC care during both FY16 and FY19.^15^ Ratings of VA and non-VA hospitals were mixed in a third study (VA: n = 135 facilities; non-VA: n = 402 facilities), with non-VA hospitals receiving higher scores for hospital quietness, pain management, responsiveness of hospital staff, and communication with doctors or nurses, and VA hospitals being rated higher for communication about medicine, hospital cleanliness, and care transitions.^8^ The fourth study (VA: n = 129 facilities; non-VA: n = 4010 facilities), which assessed national samples of VA and non-VA hospitals, also reported mixed results. Some patient experience domains were better in non-VA care, but there was no difference for other domains.^12^ In the fifth study (VA: n = 303; non-VA; n = 242), patient centeredness was not different (p = 0.243) between VA tele-mental healthcare and VA-paid, in-person mental healthcare in the community.^18^ In the last study (VA: n = 39 facilities; non-VA: n = 39 facilities), VA and non-VA university affiliate Yelp ratings did not differ after adjustment for bed size, teaching hospital and graduate medical education status, and The Joint Commission certification.^23^

### Cost/Efficiency

We identified 6 studies reporting on efficiency or cost outcomes: one study analyzed patients with cardiac disease,^11^ one study examined imaging in patients with prostate cancer,^30^ one study investigated end-of-life care,^19^ one study looked at patients undergoing dialysis,^43^ another study analyzed low-value prostate-specific antigen testing (PSA),^35^ and the last study examined in-person and tele-mental healthcare.^18^ Five studies were of good quality,^11,19,30,35,43^ and one was of fair quality.^18^ Cost/efficiency in VA care was equal or mixed to non-VA care in four studies,^11,18,19,43^ and better in two studies.^30,35^

In the first study (VA: n = 15,340; non-VA: n = 3715), costs were lower in VA than in VA-paid community care for patients receiving PCI (VA: $15,683 vs. non-VA: $22,025; p < 0.001) but higher in VA than VA-paid CC for patients receiving CABG (VA: $63,144 vs. non-VA: $55,526; p < 0.01).^11^ In a study (VA: n = 27,811; non-VA: n = 56,671) of low-risk prostate cancer patients, those receiving care in VA were less likely to receive guideline-discordant imaging (relative risk 0.79, 95% CI 0.67, 0.92) compared to those receiving care paid by Medicare.^30^ In the third study (VA: n = 10,341; non-VA: n = 18,542), dually enrolled cancer patients had similar costs of care no matter if they were either Medicare- or VA-reliant.^19^ In the fourth study (VA: n = 1101; non-VA: n = 3805 [VA Purchased Care], 18,267 [Medicare]), days of hospitalization after dialysis were similar in VA and non-VA settings.^43^ In the fifth study (VA: n = 36,469; non-VA: n = 17,981), low-value PSA testing was associated with 9.9 fewer downstream services per 100 Veterans (95% CI 9.7 to 10.1) and $11.9 less spending per Veteran, (95% CI $7.6 to $16.2) in VA compared to non-VA care.^35^ In the last study (VA: n = 303; non-VA; n = 242), the numbers of encounters did not significantly differ (p = 0.276) between patients receiving VA tele-mental healthcare or VA-paid, in-person mental healthcare in the community.^18^

### Equity

We did not find any studies reporting on equity outcomes.

## DISCUSSION

Our systematic review identified 37 studies comparing clinical quality, safety, access, patient experience, or efficiency/cost between VA delivered care and non-VA delivered care. The large majority of studies assessed clinical quality and safety; followed by comparisons of access to care. Few studies, only 6 in each category, assessed patient experience or cost/efficiency. We found no studies comparing VA to non-VA care on equity. In the domain of clinical quality and safety, the great majority of studies found that VA care is as good as, or better than, care in the community. For the domains of access and of cost/efficiency, VA care was not consistently better or worse than non-VA care. The few studies of patient experience found that VA care and non-VA were not consistently different, but VA care was never worse.

The studies best able to address implications of the Choice and MISSION acts were designed to capture data of Veterans receiving VA-paid community care. In these comparisons, clinical quality and safety was generally better in VA-delivered care. Differences between sites of care were more mixed for the other domains – access, patient experience, and cost.

Key among the clinical quality and safety outcomes is mortality. In studies of cardiac revascularization,^11^ ESRD^42^ and pre-ESRD^27^ dialysis, COPD,^28^ and ambulance rides to emergency departments,^14^ mortality was lower in VA care. In the two other studies with this outcome, mortality did not differ between VA and non-VA care.

The overarching conclusion from the published studies since 2015 reinforce the conclusions of the two prior reviews^1,2^ of studies comparing VA care to non-VA care: on average, VA care performs better than or similar to non-VA care in the domain of clinical quality and safety. This review expands those earlier conclusions to include the healthcare quality domain of access, and combined with studies from the 2016 review can now also make early conclusions on patient experience and efficiency/cost. In each of these domains of access, patient experience, and cost/efficiency, comparative results of performance are more mixed than in the domain of quality/safety, however in the domain of patient experience VA care was never worse.

### Limitations

Beyond the usual limitation of any systematic review, the quantity and quality of the original studies, we had several additional limitations. First, there was the possibility of publication bias or subconscious investigator bias, as most of the published studies were written by VA authors. We scrutinized each study for objective evidence of bias to diminish the contribution of such bias to our overall conclusions. Second, there was the possibility of confounding by patient populations in VA or non-VA care. Studies attempted to control for this through multivariable methods (e.g., Chan 2022^14^ had robust controls and concluded that post-ambulance care had lower mortality in VA), but VA patients generally have worse, unmeasured social determinants of health^45,46^ than those in the community, so included studies may favor non-VA care. Finally, we were limited by how different stakeholders may value our included outcomes. We did not attempt to rank different outcomes (e.g., wait times or provider ratings) by importance, as this assessment could differ by stakeholder.

### Future Research

Despite several dozen publications comparing VA care with non-VA care, there are several clinical areas utilized heavily in the community via the MISSION Act, such as physical medicine and rehabilitation, that are not represented in the literature. In addition, it would greatly facilitate comparisons of VA care to non-VA care if non-VA care had the same degree of comprehensive performance data that are publicly available. Lastly, we expect that this topic is a moving target, and thus this review needs regular updating of published studies to remain up to date.

## CONCLUSION

In general, most published studies of comparisons of healthcare quality show that Veterans getting care from VA get the same or better clinical quality than Veterans getting community care or the general public getting non-VA care.

## Supplementary Information

Below is the link to the electronic supplementary material.

Supplementary file1 (DOCX 233 KB)

## Data Availability

The data supporting the findings of this study are available within the article and its supplementary material.

## Declarations

### Disclaimer

The views expressed are those of the authors and do not represent the views of the US Department of Veterans Affairs or the United States Government.
