# Supplementary material for: Veterans Health Administration (VA) vs. Non-VA Healthcare Quality: A Systematic Review
Source: J Gen Intern Med. 2023 Apr 19;38(9):2179–88. doi: 10.1007/s11606-023-08207-2 (PMC10361919; doi:10.1007/s11606-023-08207-2)
Supplement: Supplementary file 1 — Supplementary file1 (DOCX 233 KB) [file 11606_2023_8207_MOESM1_ESM.docx]

**Supplementary Material**

**Supplementary Material 1: Search strategies**

PubMed

English; 2015 to Present

Search run: 9 March 2023

"United States Department of Veterans Affairs"[mh] OR "Veterans Health"[mh] OR "veterans health services"[mh] OR "Hospitals, Veterans"[mh] OR "veterans affairs"[tiab] OR "veterans health"[tiab] OR "veterans choice"[tiab]

AND

Compar*[ti] OR "vs"[ti] OR versus[ti] OR difference[ti] OR "dually enrolled"[tiab] OR "dual system*"[tiab] OR "dual enrollment"[tiab] OR "overlapping use"[tiab] OR (examine*[tiab] AND (access*[tiab] OR availab*[tiab])) OR (("community care*"[tiab] OR "Community Health Services"[Majr]) AND impact*[tiab]) OR ((other[tiab] OR "private sector"[tiab] OR "non-VA"[tiab] OR medicare[tiab] OR "commercially managed"[tiab] OR "non veteran*"[tiab] OR "non VAMC"[tiab] OR "non va"[tiab] OR "non federal hospital*"[tiab] OR "university hospital*"[tiab] OR nonveteran*[tiab] OR "nonfederal hospital*"[tiab]) AND (compar*[tiab] OR comparative study[pt]))

Results: 1826

PubMed Update

English; December 2021 to Present

Search run: 9 March 2023

"United States Department of Veterans Affairs"[mh] OR "Veterans Health"[mh] OR "veterans health services"[mh] OR "Hospitals, Veterans"[mh] OR "veterans affairs"[tiab] OR "veterans health"[tiab] OR "veterans choice"[tiab]

AND

Compar*[ti] OR "vs"[ti] OR versus[ti] OR difference[ti] OR "dually enrolled"[tiab] OR "dual system*"[tiab] OR "dual enrollment"[tiab] OR "overlapping use"[tiab] OR (examine*[tiab] AND (access*[tiab] OR availab*[tiab])) OR (("community care*"[tiab] OR "Community Health Services"[Majr]) AND impact*[tiab]) OR ((other[tiab] OR "private sector"[tiab] OR "non-VA"[tiab] OR medicare[tiab] OR "commercially managed"[tiab] OR "non veteran*"[tiab] OR "non VAMC"[tiab] OR "non va"[tiab] OR "non federal hospital*"[tiab] OR "university hospital*"[tiab] OR nonveteran*[tiab] OR "nonfederal hospital*"[tiab]) AND (compar*[tiab] OR comparative study[pt]))

Results: 374

APA PsycINFO

English; 2015 to Present

Publication Limiter: Journal Articles

Search run: 10 March 2023

TI("Veterans Health" OR "veterans affairs" OR "veterans health" OR "veterans choice") OR AB("Veterans Health" OR "veterans affairs" OR "veterans health" OR "veterans choice")

AND

TI(Compar* OR "vs" OR versus OR difference) OR (TI("dually enrolled" OR "dual system*" OR "dual enrollment" OR "overlapping use") OR AB("dually enrolled" OR "dual system*" OR "dual enrollment" OR "overlapping use")) OR (TI(examine*) AND TI(access* OR availab*)) OR (TI(examine*) AND AB(access* OR availab*)) OR (AB(examine*) AND TI(access* OR availab*)) OR (AB(examine*) AND AB(access* OR availab*)) OR ((TI("community care*") OR AB("community care*") OR MM("Community Mental Health Services")) AND (TI(impact* OR AB(impact*))) OR (TI(other OR "private sector" OR "non-VA" OR medicare OR "commercially managed" OR "non veteran*" OR "non VAMC" OR "non va" OR "non federal hospital*" OR "university hospital*" OR nonveteran* OR "nonfederal hospital*") OR AB(other OR "private sector" OR "non-VA" OR medicare OR "commercially managed" OR "non veteran*" OR "non VAMC" OR "non va" OR "non federal hospital*" OR "university hospital*" OR nonveteran* OR "nonfederal hospital*")) AND (TI(compar*) OR AB(compar*) OR TI("comparative study")))

Results: 112

Web of Science

English; 2015 to Present

Search run: 15 March 2023

TI=("veterans affairs" OR "veterans health" OR "veterans choice" OR "veterans hospital") OR AB=("veterans affairs" OR "veterans health" OR "veterans choice" OR "veterans hospital")

AND

TI=(compar* OR "vs" OR versus OR difference) OR TI=("dually enrolled" OR "dual system*" OR "dual enrollment" OR "overlapping use") OR AB=("dually enrolled" OR "dual system*" OR "dual enrollment" OR "overlapping use") OR ((TI=(examine*) OR AB=(examine*)) AND (TI=(access* OR availab*) OR AB=(access* OR availab*))) OR ((TI=("community care*") OR AB=("community care*")) AND (TI=(impact*) OR AB=(impact*))) OR (TI=(other OR "private sector" OR "non-VA" OR medicare OR "commercially managed" OR "non veteran*" OR "non VAMC" OR "non va" OR "non federal hospital*" OR "university hospital*" OR nonveteran* OR "nonfederal hospital*") OR AB=(other OR "private sector" OR "non-VA" OR medicare OR "commercially managed" OR "non veteran*" OR "non VAMC" OR "non va" OR "non federal hospital*" OR "university hospital*" OR nonveteran* OR "nonfederal hospital*")) AND (TI=(compar*) OR AB=(compar*))

Results: 136

Total: 2448

Total after deduplication: 2415

**Supplementary Material 2: Study selection, data abstraction, and study quality assessment**

*Study selection criteria*

Studies were included at either the abstract or the full-text level if they were original research studies of any design and made comparisons in one of two ways: (1) Veterans receiving VA care versus Veterans receiving non-VA care (community care [CC]) or (2) Veterans receiving VA care versus the general population of non-Veteran patients receiving non-VA care in the community (non-VA). We included as healthcare quality any outcomes within the Institute of Medicine 6 domains of health care: clinical quality, safety, access, patient experience, efficiency (cost) and equity.^1^ The first review of VA vs. non-VA care in 2011 only examined clinical quality and safety,^2^ and the second review in 2016^3^ added the four additional domains, but found relatively few articles per domain, and did not discuss these additional domains in detail.

*Data abstraction items*

We abstracted information on medical condition, type of outcome reported, populations under comparison, years of data were collected, sample size, years of data collected, control variables, outcomes, findings, and statistical methods and elements of study quality (whether study years were contemporaneous; sampling approach; geographic representativeness; similarity of outcomes between the comparison groups).

*Study quality assessment criteria*

For this review we adapted the 6 items originally used in the 2011 and 2016 reviews^2,3^ to the following criteria: (1) whether the time frames for the measurement were contemporaneous for both groups (levels: [A] contemporaneous; [B] all between A and C; [C]: non-contemporaneous) (2) whether the samples were national or representative for both groups (levels: [A] representative or national samples (both VA and non-VA); [B] all between A and C; [C]: small, limited, unequal or non-representative samples) (3) whether the study quality measures used to assess care in both groups were identical or nearly identical (levels: [A] identical; [B]: sufficiently similar for valid comparison; [C]: sufficiently dissimilar to present a threat to valid comparison); and (4) whether the analysis had enough sample size and appropriate statistical methods to test the hypotheses (levels: [A] sufficient sample size and/or methods appropriate to address hypothesis[ses]; [B]: all between A and C; [C]: insufficient sample size and/or methods questionable to address hypothesis[ses]). Studies were rated for each criterion on a 3-level scale from A (highest) to C (lowest). Studies fully meeting all of these criteria at the highest level were considered to be of good study quality and given greater weight than studies meeting some criteria at the middle level, which were considered to be of fair study quality. Studies rated at the lowest level for one or more criteria were consider of poor study quality and removed from the analysis.

**Supplementary Material 3: Fair quality studies**

Two of these studies analyzed preexisting samples from clinical trials.^4,5^ Three studies had very unbalanced samples; either VA or non-VA groups were much smaller than the others.^6-8^ Two studies had balanced, but small samples, and the latter study additional only analyzed data from one site and did not adjust for patient characteristics in their models.^9,10^ Heidenreich and colleagues only analyzed the Yelp ratings of 39 VA hospitals (out of a possible 131) and their university affiliates due to the lack of reviews of the remaining facilities.^11^ Mody et al. only had data on VA and non-VA nursing homes from approximately half of all states.^12^ Another study by Axon and colleagues only analyzed VA and non-VA facilities in the state of South Carolina.^13^ Shields and colleagues were not able to adjust for patient characteristics in their analysis of clinical quality of inpatient psychiatric care, so different patient populations between VA and non-VA facilities may have biased their results.^14^ Presley and colleagues also did not adjust for patient characteristics in their analysis of aggressive end of life care for nonsmall lung cancer, and the composition of their multi-component outcome was unclear.^15^

**Supplementary Material 4: Excluded studies**

Does not compare quality of clinical data in VA and US non-VA Settings, n=51

1. Augustine, M.R., et al., Reasons Older Veterans Use the Veterans Health Administration and Non-VHA Care in an Urban Environment. J Am Board Fam Med, 2021. 34(2): p. 291-300.

2. Benzer, J.K., et al., Survey of Patient-Centered Coordination of Care for Diabetes with Cardiovascular and Mental Health Comorbidities in the Department of Veterans Affairs. Journal of General Internal Medicine, 2019. 34(1): p. 43-49.

3. Bouldin, E.D., et al., Medicare-VHA dual use is associated with poorer chronic wound healing. Wound Repair Regen, 2016. 24(5): p. 913-922.

4. Burke, J.F. and B.C. Callaghan, Author response: Neuroimaging overuse is more common in Medicare compared with the VA. Neurology, 2017. 88(6): p. 608.

5. Dayoub, E.J., et al., Federal Payments for Coronary Revascularization Procedures Among Dual Enrollees in Medicare Advantage and the Veterans Affairs Health Care System. JAMA Netw Open, 2020. 3(4): p. e201451.

6. Desmarais, J. and C.Q. Chu, Utility of Anakinra in Acute Crystalline Diseases: A Retrospective Study Comparing a University Hospital with a Veterans Affairs Medical Center. J Rheumatol, 2019. 46(7): p. 748-750.

7. Feyman, Y., A. Legler, and K.N. Griffith, Appointment wait time data for primary & specialty care in veterans health administration facilities vs. community medical centers. Data Brief, 2021. 36: p. 107134.

8. Gidwani-Marszowski, R., et al., Quality Of End-Of-Life Care Is Higher In The VA Compared To Care Paid For By Traditional Medicare. Health Aff (Millwood), 2018. 37(1): p. 95-103.

9. Griebling, T.L., Re: Comparing Catheter-Associated Urinary Tract Infection Prevention Programs between Veterans Affairs Nursing Homes and Non-Veterans Affairs Nursing Homes. J Urol, 2018. 200(6): p. 1142.

10. Hebert, P.L., et al., Reliance on Medicare Providers by Veterans after Becoming Age-Eligible for Medicare is Associated with the Use of More Outpatient Services. Health Serv Res, 2018. 53 Suppl 3(Suppl Suppl 3): p. 5159-5180.

11. Johnston, J.C. and T.P. Sartwelle, Letter re: Neuroimaging overuse is more common in Medicare compared with the VA. Neurology, 2017. 88(6): p. 608.

12. Jones, A.L., et al., National Media Coverage of the Veterans Affairs Waitlist Scandal: Effects on Veterans' Distrust of the VA Health Care System. Med Care, 2021. 59(Suppl 3): p. S322-S326.

13. Leonard, C., et al., Operationalizing an Implementation Framework to Disseminate a Care Coordination Program for Rural Veterans. Journal of General Internal Medicine, 2019. 34(1): p. 58-66.

14. Lewinski, A.A., et al., Applied Rapid Qualitative Analysis to Develop a Contextually Appropriate Intervention and Increase the Likelihood of Uptake. Med Care, 2021. 59(Suppl 3): p. S242-S251.

15. Loganathan, S.K., et al., Racial and Ethnic Differences in Satisfaction with Care Coordination Among VA and non-VA Medicare Beneficiaries. Health Equity, 2017. 1(1): p. 50-60.

16. Machlin, S.R. and P. Muhuri, Characteristics and Health Care Expenditures of VA Health System Users versus Other Veterans, 2014-2015 (Combined), in Statistical Brief (Medical Expenditure Panel Survey (US)). 2001, Agency for Healthcare Research and Quality (US): Rockville (MD).

17. Malhotra, A., M. Vaughan-Sarrazin, and G.E. Rosenthal, Elderly veterans with dual eligibility for VA and Medicare services: where do they obtain a colonoscopy? Am J Manag Care, 2015. 21(4): p. e264-70.

18. Mattocks, K.M., et al., Understanding Maternity Care Coordination for Women Veterans Using an Integrated Care Model Approach. Journal of General Internal Medicine, 2019. 34(1): p. 50-57.

19. McCreight, M.S., et al., Practical Use of Process Mapping to Guide Implementation of a Care Coordination Program for Rural Veterans. Journal of General Internal Medicine, 2019. 34(1): p. 67-74.

20. Mohr, D.C., et al., Organizational Coordination and Patient Experiences of Specialty Care Integration. Journal of General Internal Medicine, 2019. 34(1): p. 30-36.

21. Mudumbai, S.C., et al., Perioperative Opioid Prescribing Patterns and Readmissions After Total Knee Arthroplasty in a National Cohort of Veterans Health Administration Patients. Pain Med, 2020. 21(3): p. 595-603.

22. Nadpara, P.A., et al., Risk Factors for Serious Prescription Opioid-Induced Respiratory Depression or Overdose: Comparison of Commercially Insured and Veterans Health Affairs Populations. Pain Med, 2018. 19(1): p. 79-96.

23. Nelson, R.E., et al., The Impact of a Change in the Price of VA Health Care on Utilization of VA and Medicare Services. Med Care, 2018. 56(7): p. 569-576.

24. Nelson, R.E., et al., Costs Associated with Health Care Services Accessed through VA and in the Community through Medicare for Veterans Experiencing Homelessness. Health Serv Res, 2018. 53 Suppl 3(Suppl Suppl 3): p. 5352-5374.

25. Noël, P.H., et al., Patient experience of health care system hassles: Dual-system vs single-system users. Health Serv Res, 2020. 55(4): p. 548-555.

26. Nuti, S.V., L. Qin, and H.M. Krumholz, Outcome After Admission at Veterans Affairs vs Non-Veterans Affairs Hospitals--Reply. Jama, 2016. 316(3): p. 346.

27. O'Hanlon, C.E., C. Farmer, and C. Gidengil, Comparing VA to Non-VA Care. J Gen Intern Med, 2017. 32(2): p. 152.

28. Olmos-Ochoa, T.T., et al., Staff Perspectives on Primary Care Teams as De Facto “Hubs” for Care Coordination in VA: a Qualitative Study. Journal of General Internal Medicine, 2019. 34(1): p. 82-89.

29. Pershing, S., et al., Treating age-related macular degeneration: comparing the use of two drugs among medicare and veterans affairs populations. Health Aff (Millwood), 2015. 34(2): p. 229-38.

30. Peterson, K., et al., Health Care Coordination Theoretical Frameworks: a Systematic Scoping Review to Increase Their Understanding and Use in Practice. Journal of General Internal Medicine, 2019. 34(1): p. 90-98.

31. Radomski, T.R., M.J. Fine, and W.F. Gellad, Outcome After Admission at Veterans Affairs vs Non-Veterans Affairs Hospitals. Jama, 2016. 316(3): p. 345-6.

32. Radomski, T.R., et al., The Impact of Medication-Based Risk Adjustment on the Association Between Veteran Health Outcomes and Dual Health System Use. J Gen Intern Med, 2017. 32(9): p. 967-973.

33. Ramkumar, M. and S.T. Crowley, Kidney Transplantation Rates of Veterans Administration-Listed Patients Compared with Rates of Patients on Nonveteran Lists. J Am Soc Nephrol, 2018. 29(10): p. 2449-2450.

34. Rinne, S.T., et al., VA Provider Perspectives on Coordinating COPD Care Across Health Systems. Journal of General Internal Medicine, 2019. 34(1): p. 37-42.

35. Rose, D.E., et al., Variations in VA and Medicare Use Among Veterans With Diabetes: Impacts on Ambulatory Care Sensitive Conditions Hospitalizations for 2008, 2009, and 2010. Med Care, 2019. 57(6): p. 425-436.

36. Rose, L., et al., Association of Expanded Health Care Networks With Utilization Among Veterans Affairs Enrollees. JAMA Netw Open, 2021. 4(10): p. e2131141.

37. Rosenberg, K., End-Of-Life Cancer Care For Veterans Through The VA Vs. Medicare. Am J Nurs, 2018. 118(5): p. 70.

38. Trivedi, A.N., et al., Dual Use and Hospital Admissions among Veterans Enrolled in the VA's Homeless Patient Aligned Care Team. Health Serv Res, 2018. 53 Suppl 3(Suppl Suppl 3): p. 5219-5237.

39. Trivedi, A.N., et al., Agreement Between HEDIS Performance Assessments in the VA and Medicare Advantage: Is Quality in the Eye of the Beholder? Inquiry, 2016. 53.

40. Tummalapalli, S.L. and S. Keyhani, Trends in Preventative Health Services for Veterans with Military Coverage Compared to Non-Military Coverage. J Gen Intern Med, 2020. 35(4): p. 1330-1333.

41. Valle, J.A., et al., Dual antiplatelet therapy in non-ST elevation acute coronary syndromes at Veterans Affairs Hospitals. Heart, 2019. 105(20): p. 1575-1582.

42. Veet, C.A., et al., Impact of Healthcare Delivery System Type on Clinical, Utilization, and Cost Outcomes of Patient-Centered Medical Homes: a Systematic Review. J Gen Intern Med, 2020. 35(4): p. 1276-1284.

43. Ward, R., et al., An Evaluation of Statin Use Among Patients with Type 2 Diabetes at High Risk of Cardiovascular Events Across Multiple Health Care Systems. J Manag Care Spec Pharm, 2020. 26(9): p. 1090-1098.

44. Weeks, W.B., Comparing VA to Non-VA Care. J Gen Intern Med, 2017. 32(2): p. 150-151.

45. Wong, E.S., et al., Impact of VHA's primary care intensive management program on dual system use. Healthc (Amst), 2020. 8(3): p. 100450.

46. Wray, C.M., M. Khare, and S. Keyhani, Access to Care, Cost of Care, and Satisfaction With Care Among Adults With Private and Public Health Insurance in the US. JAMA Netw Open, 2021. 4(6): p. e2110275.

47. Wray, C.M., L. Lopez, and S. Keyhani, "Comparing VA and Non-VA Care Quality". J Gen Intern Med, 2019. 34(4): p. 485.

48. Yoon, J., et al., Use of the Veterans' Choice Program and Attrition From Veterans Health Administration Primary Care. Med Care, 2020. 58(12): p. 1091-1097.

49. Yu, M.K., et al., Trends in Timing of Dialysis Initiation within Versus Outside the Department of Veterans Affairs. Clin J Am Soc Nephrol, 2015. 10(8): p. 1418-27.

50. Zulman, D.M., et al., Effects of Intensive Primary Care on High-Need Patient Experiences: Survey Findings from a Veterans Affairs Randomized Quality Improvement Trial. Journal of General Internal Medicine, 2019. 34(1): p. 75-81.

51. Gaffney, A., et al., Uptake and Equity in Influenza Vaccination Among Veterans with VA Coverage, Veterans Without VA Coverage, and Non-Veterans in the USA, 2019-2020. J Gen Intern Med, 2022: p. 1-8.

No Outcomes of Interest, n=2

1. Dismuke-Greer, C.E., et al., Economic impact of comorbid TBI-dementia on VA facility and non-VA facility costs, 2000-2020. Brain Inj, 2022. **36**(5): p. 673-682.

2. Wray, C., et al., Digital Health Skillsets and Digital Preparedness: Comparison of Veterans Health Administration Users and Other Veterans Nationally. JMIR Form Res, 2022. **6**(1): p. e32764.

Not research, n=7

1. Cordasco, K.M., et al., Coordinating Care Across VA Providers and Settings: Policy and Research Recommendations from VA’s State of the Art Conference. Journal of General Internal Medicine, 2019. 34(1): p. 11-17.

2. Cordasco, K.M., et al., Improving Care Coordination for Veterans Within VA and Across Healthcare Systems. Journal of General Internal Medicine, 2019. 34(1): p. 1-3.

3. Gittell, J.H. and L. Hajjar, Strengthening Patient-Centered Care in the VHA: A Relational Model of Change. Journal of General Internal Medicine, 2019. 34(1): p. 7-10.

4. Mattocks, K.M., et al., Recommendations for the Evaluation of Cross-System Care Coordination from the VA State-of-the-art Working Group on VA/Non-VA Care. Journal of General Internal Medicine, 2019. 34(1): p. 18-23.

5. Mattocks, K.M., et al., Innovations in Community Care Programs, Policies, and Research. Med Care, 2021. 59(Suppl 3): p. S229-S231.

6. McDonald, K.M., et al., Incorporating Theory into Practice: Reconceptualizing Exemplary Care Coordination Initiatives from the US Veterans Health Delivery System. Journal of General Internal Medicine, 2019. 34(1): p. 24-29.

7. Mengeling, M.A., et al., Partnership Forum: The Role of Research in the Transformation of Veterans Affairs Community Care. Med Care, 2021. 59(Suppl 3): p. S232-S241.

Background, n=6

1. Garvin, L.A., et al., Interorganizational Care Coordination of Rural Veterans by Veterans Affairs and Community Care Programs: A Systematic Review. Med Care, 2021. 59(Suppl 3): p. S259-S269.

2. Gordon, S.H., et al., County-level Predictors of Growth in Community-based Primary Care Use Among Veterans. Med Care, 2021. 59(Suppl 3): p. S301-S306.

3. Greenstone, C.L., et al., Standardizing Care Coordination Within the Department of Veterans Affairs. Journal of General Internal Medicine, 2019. 34(1): p. 4-6.

4. Hynes, D.M., et al., Veterans' Use of Veterans Health Administration Primary Care in an Era of Expanding Choice. Med Care, 2021. 59(Suppl 3): p. S292-S300.

5. Mattocks, K.M., et al., Understanding VA's Use of and Relationships With Community Care Providers Under the MISSION Act. Med Care, 2021. 59(Suppl 3): p. S252-S258.

6. Vashi, A.A., et al., Community Urgent Care Use Following Implementation of the Veterans Affairs Maintaining Internal Systems and Strengthening Integrated Outside Networks Act. Med Care, 2021. 59(Suppl 3): p. S314-S321.

About surgery, n=7

1. Billig, J.I., et al., The Impact of Community Care Referral on Time to Surgery for Veterans With Carpal Tunnel Syndrome. Med Care, 2021. 59(Suppl 3): p. S279-S285.

2. George, E.L., et al., Comparing Veterans Affairs and Private Sector Perioperative Outcomes After Noncardiac Surgery. JAMA Surg, 2021.

3. Harris, A.H.S., et al., Comparing Complication Rates After Elective Total Knee Arthroplasty Delivered Or Purchased By The VA. Health Aff (Millwood), 2021. 40(8): p. 1312-1320.

4. Pettey, W.B.P., et al., Comparing Driving Miles for Department of Veterans Affairs-delivered Versus Department of Veterans Affairs-purchased Cataract Surgery. Med Care, 2021. 59(Suppl 3): p. S307-S313.

5. Rosen, A., et al., Comparing Postoperative Readmission Rates between Veterans Receiving Total Knee Arthroplasty in the Veterans Health Administration (VA) versus Community Care. Medical Care, 2021.

1. Dizon, M.P., et al., Comparing the Quality of Ambulatory Surgical Care for Skin Cancer in a Veterans Affairs Clinic and a Fee-For-Service Practice Using Clinical and Patient-Reported Measures. PLoS One, 2017. 12(1): p. e0171253.
2. Geraci, T., et al., Lobectomy for Lung Cancer at Veterans Administration Medical Center Versus Academic Medical Center. Ann Thorac Surg, 2017. 103(6): p. 1715-1722.

Poor study quality, n=5

1. Bartel, M.J., D.J. Robertson, and H. Pohl, Colonoscopy practice for veterans within and outside the Veterans Affairs setting: a matched cohort study. Gastrointest Endosc, 2016. 84(2): p. 272-8.

2. Chao, D., et al., Outcomes Comparison of the Veterans' Choice Program With the Veterans Affairs Health Care System for Hepatitis C Treatment. Fed Pract, 2020. 37(Suppl 3): p. S18-s24.

3. Cullen, S.W., et al., Comparing Rates of Adverse Events and Medical Errors on Inpatient Psychiatric Units at Veterans Health Administration and Community-based General Hospitals. Med Care, 2019. 57(11): p. 913-920.

4. Dueker, J.M. and A. Khalid, Performance of the Veterans Choice Program for Improving Access to Colonoscopy at a Tertiary VA Facility. Fed Pract, 2020. 37(5): p. 224-228.

5. Grubbs, K.M., et al., A Comparison of Collaborative Care Outcomes in Two Health Care Systems: VA Clinics and Federally Qualified Health Centers. Psychiatr Serv, 2018. 69(4): p. 431-437.

**Supplementary Material 5: Evidence table for included studies**

| **Author,  Year of publication  Large Database** (y/n) **Study Design Medical condition**  **Outcome domains** | **Years of source data,**   **Comparison of VA veterans to: __;**   **Data source(s)** | **VA Care:  n Outcomes - raw values** | **Non-VA Care:  n (population) Outcomes - raw value** | **Comparison statistics adjusted model findings** | **Statistical method other methods of controlling;**  **covariates in model** | **Bias Criteria met?** | **Comments & Reason if bias criteria not met** |
| --- | --- | --- | --- | --- | --- | --- | --- |
| Nuti,  2016^16^  Y (**National**) Retrospective  Acute myocardial infarction, heart failure, pneumonia  Clinical quality/safety | 2013-2016, vs. other non-VA; CMS Standard Analytic Files and Enrollment Database vs. VA administrative claims | n: 7929-26,231  Mortality (AMI): M 13.52/30d, 95% CI 13.38 to 13.66; Mortality (HF): M 11.43/30d, 95% CI 11.11 to 11.75;  Mortality (Pneu): M 12.63/30d, 95% CI 12.19 to 13.07;  Readmissions (AMI): M 17.84/30d, 95% CI 17.71 to 17.96;  Readmissions (HF): M 24.66/30d, 95% CI 24.31 to 25.02;  Readmissions (Pneu):  M 19.44/30d, 95% CI 19.19 to 19.69 | n: 124,220-269,856  Mortality (AMI): M 13.69/30 d, 95% CI 13.64 to 13.74;  Mortality (HF): M 11.87/30d, 95% CI 11.80 to 11.93;  Mortality (Pneu): M 12.17/30d, 95% CI 12.08 to 12.26;  Readmissions (AMI): M 17.21/30d; 95% CI 17.17 to 17.25;  Readmissions (HF): M 23.46/30d; 95% CI 23.39 to 23.53;  Readmissions (Pneu): M 18.68/30d; 95%CI 18.63 to 18.73 | Mortality (AMI): VA<non-VA,p=0.02;  Mortality (HF): VA<non-VA, p=0.008;  Mortality (Pneu): VA>non-VA, p=0.045;  Readmissions (AMI): VA>non-VA, p<0.001;  Readmissions (HF): VA>non-VA, p<0.001;  Readmisions (Pneu): VA>non-VA, p<0.001; | Statistics: Hierarchical logistic regression to estimate values; t-tests to compare  Other methods of controlling: NR  Covariates: Age, patient cardiovascular medical history, comorbid conditions, hospital random effects | Y |  |
| Vanneman, 2020^17^  Y (**National**)  Retrospective  Outpatient specialty, primary, and mental health care  Access  Patient experience | 2016-2017, vs. Veterans in VA-paid community care; SHEP vs. CAHPS | n=29,095-432,218 (combined VA and non-VA)  NR | n=29,095-432,218 (combined VA and non-VA)  NR | Access to care (specialty care):  -0.0023 (VA vs. non-VA; p=ns);  Access to care (primary care):  -0.0003 (VA vs. non-VA; p=ns);  Access to care (mental health):  -0.001  Patient experience (specialty care): 0.0005 (VA vs. non-VA; p=ns);  Patient experience (primary care):  -0.0137 (VA vs. non-VA; p=ns);  Patient experience (mental health):  -0.0218 (VA vs. non-VA; p=ns); | Statistics: Multivariate regression models  Other methods of controlling: NR  Covariates: age, sex, race, ethnicity, education level, marital status, rurality, VA enrollment priority, and Nosos health risk score, perceived physical health status, perceived mental health status, insurance status, number of days between the outpatient visit and survey return date, and VA facility fixed effects | Y | Regression coefficients over entire time period reported |
| Gurewich, 2021^18^  Y (**National**)  Retrospective  Physical therapy, cardiology, optometry, dental care, and orthopedics  Access | 2014-2018 (FY15-FY18), vs. Veterans in VA-paid community care; CDW (both VA and non-VA) | n=420,590 (FY15), 487,014 (FY18)  FY15 (wait time in days for urban Veterans):  Physical therapy: 30.62  Cardiology: 26.77  Optometry: 42.84  Orthopedic: 35.26  Dental: 27.70;  FY18 (wait time in days for urban Veterans):  Physical therapy: 26.26  Cardiology: 24.15  Optometry: 34.32  Orthopedic: 27.73  Dental: 24.01; | n=76,706 (FY15), 150,429 (FY18)  FY15 (wait time in days for urban Veterans):  Physical therapy: 28.94  Cardiology: 28.46  Optometry: 41.85  Orthopedic: 37.35  Dental: 25.99;  FY18 (wait time in days for urban Veterans):  Physical therapy: 28.84  Cardiology: 27.55  Optometry: 36.90  Orthopedic: 32.87  Dental: 25.90; | VA had greater wait time declines from FY15 to FY18 than non-VA except for cardiology (p<0.001) | Statistics: Linear regression  Other methods of controlling: NR  Covariates: Rurality, age, sex, race/ethnicity, marital status, FY, Nosos score, priority level, age/sex*FY18 interactions | Y | NA |
| Davila, 2021^19^  Y (**National**)  Retrospective  Primary and specialty care  Access  Patient experience | FY16-FY19, vs. Veterans in VA-paid community care; SHEP and CDW (both VA and non-VA) | n=1,019,732  FY16 (primary care, access, urban): 3.18;  FY16 (specialty care, access, urban): 3.09;  FY19 (primary care, access, urban): 3.27;  FY19 (specialty care, access, urban): 3.17;  FY16 (primary care, access, rural): 3.24;  FY16 (specialty care, access, rural): 3.15;  FY19 (primary care, access, rural): 3.31;  FY19 (specialty care, access, rural): 3.23 ;  FY16 (primary care, provider rating, urban): 8.83;  FY16 (specialty care, provider rating, urban): 8.69;  FY19 (primary care, provider rating, urban): 8.92;  FY19 (specialty care, provider rating, urban): 8.88;  FY16 (primary care, provider rating, rural): 8.80;  FY16 (specialty care, provider rating, rural): 8.73;  FY19 (primary care, provider rating, rural): 8.90;  FY19 (specialty care, provider rating, rural): 8.92 | n=63,638  FY16 (primary care, access, urban): 2.91;  FY16 (specialty care, access, urban): 3.17;  FY19 (primary care, access, urban): 3.12;  FY19 (specialty care, access, urban): 3.28;  FY16 (primary care, access, rural): 3.11;  FY16 (specialty care, access, rural): 3.17;  FY19 (primary care, access, rural): 3.16;  FY19 (specialty care, access, rural): 3.28;  FY16 (primary care, provider rating, urban): 7.28;  FY16 (specialty care, provider rating, urban): 8.46;  FY19 (primary care, provider rating, urban): 8.30;  FY19 (specialty care, provider rating, urban): 8.70;  FY16 (primary care, provider rating, rural): 8.14;  FY16 (specialty care, provider rating, rural): 8.43;  FY19 (primary care, provider rating, rural): 8.56;  FY19 (specialty care, provider rating, rural): 8.72 | FY 16 VA vs. CC (rural, primary care, access): 0.17;  FY 19 VA vs. CC (rural, primary care, access): 0.21;  FY 16 VA vs. CC (rural, specialty care, access):  -0.02;  FY 19 VA vs. CC (rural, specialty care, access):  -0.07;  FY 16 VA vs. CC (rural, primary care, provider rating): 0.35;  FY 19 VA vs. CC (rural, primary care, provider rating): 0.19;  FY 16 VA vs. CC (rural, specialty care, provider rating): 0.16;  FY 19 VA vs. CC (rural, specialty care, provider rating): 0.12 | Statistics: Multiple regression models  Other methods of controlling: NR  Covariates: Age, sex, race, education level, marital status, VA enrollment priority, Nosos risk score, and self-rated physical and mental health | Y | SHEP scores analyzed in raw column, effect sizes reported in comparison column; "Effect sizes [ESs] of 0.10 are often interpreted as indicating 'negligible' differences between groups; ESs of 0.20, 0.50, and 0.80 are considered 'small,' 'medium,' and 'large,' respectively" |
|  |  |  |  |  |  |  |  |
| Intrator, 2021^20^  Y (**National**)  Retrospective  Nursing homes  Clinical quality/safety | 2015-2016,  vs. non-Veterans in non-VA nursing homes; Vets and non-Vets in MDS, VA data (unspecified), and Medicare claims | n=23,839  Rehospitalization: M 22.51, SD 6.17;  Emergency department visits:  M 8.27, SD 4.56;  Successful discharge:  M 67.74, SD 11.47 | n=1,674,578  Rehospitalization: M 21.10 SD,5.94;  Emergency department visits: M 11.85, SD 5.32;  Successful discharge: M 57.04, SD 10.54 | Rehospitalization: VA>non-VA, p<0.001;  Emergency department visits: VA<non-VA, p<0.001;  Successful discharge: VA>non-VA, p<0.001 | Statistics: 2-sample z test  Other methods of controlling: NR  Covariates: CMS risk adjust model, including age, marital status, length of stay, medication utilization, treatments, comorbidities, and activities of daily living | Y | NA |
| LaBedz, 2021^21^  Y (**National**)  Retrospective  COPD  Clinical quality/safety | 2015-2018, vs. all patients in non-VA hospitals; CMS Hospital Compare (VA vs. non-VA) | n=126  Readmissions: M 15.3, standard error (SE) 0.17;  Mortality: M 6.0, SE 0.11 | n=3523  Readmissions: M 19.5 SE, 0.2;  Mortality: M 8.5 SE, 0.02 | Readmissions: VA<non-VA,  M -4.2, 95% CI -4.5 to -3.9;  Mortality: VA<non-VA,  M -2.6, 95% CI -2.8 to -2.4 | Statistics: T-tests, linear regression  Other methods of controlling: NR  Covariates: Age, comorbid conditions, and indicators of frailty | Y | Supplementary analyses: Increased readmission were associated with lower mortality for non-VA hospitals (p=0.003; “50 fewer deaths per 1000 more readmissions”); no association was found for VA hospitals |
| Gidwani, 2021^22^  Y (**National**)  Retrospective  Cancer  Cost/efficiency | FY10-FY14, vs. Veterans in non-VA hospitals; VA administrative data vs. Medicare claims | n=10,341  NR | n=18,542  NR | Total costs: VA<Medicare; beta-coeff:  M -0.1, 95% CI  -0.15 to -0.06;  Inpatient costs: VA<Medicare; beta-coeff:  M -0.12, 95% CI  -0.22 to -0.02;  Outpatient costs: VA<Medicare; beta-coeff:  M -0.31, 95% CI -0.35 to -0.28;  Drug costs: VA>Medicare; beta-coeff: M  -0.71, 95% CI 0.64 to 0.78 | Statistics: Generalized estimating equations  Other methods of controlling: Three-level models  Covariates: Age, race, distance from VA facility, rurality, enrollment priority, and type of solid tumor, and conditioning on geographic region | Y | NA |
| Griffith, 2020^23^  Y (**National**)  Retrospective  Cardiology, gastroenterology, orthopedics, and urology  Access | 2018-2019, vs. Veterans in VA-paid community care; VA CDW (for VA and non-VA) | n=2,504,355 consultations  Cardiology: M 33d, SD 8.7d;  Gastroenterology: M 53.9 SD 15.9d;  Orthopedics: M 36.2d SD 9.3d;  Urology: M 36.1d SD 9.5d;  Overall: M 41.1d SD 15.9d | n=533,609 consultations  Cardiology: M 38.0d, SD 9.2d;  Gastroenterology: M 60.3d SD 16.0d;  Orthopedics: M 43.6d SD 12.9d;  Urology: M 50.5d SD 14.5d;  Overall: M 49.0d SD 15.5d | NR | NR | Y | >50% of VA facilities had lower wait times for cardiology, orthopedics, urology, and overall |
| Gidwani-Marszowski, 2020^24^  Y (**National**)  Retrospective  Cancer  Clinical quality/safety | FY10-FY14, vs. Veterans in non-VA care; VA and Medicare administrative data | n=9522  444 potentially avoidable hospitalizations | n=17,921  1271 potentially avoidable hospitalizations | Medicare vs. VA: adjusted odds ratio 1.55, 95% CI 1.37 to 1.66 | Statistics: Generalized estimating equations with  a logit link and a binomial family  Other methods of controlling: Patients nested within geographic area (hospital referral region)  Covariates: Age, number of chemotherapy treatments, receipt of concurrent radiotherapy (defined as radiotherapy within 14 days of the receipt of chemotherapy), and cancer type | Y | Sensitivity analysis covariates: enrollment priority, race, rurality, and distance from a VA facility |
| Penn, 2019^25^  Y (**National**)  Retrospective  Primary care, dermatology, cardiology,  orthopedics  Access | 2014-2017, vs. non-Veterans in non-VA community care; VA administrative data vs. Merritt Hawkins secret shopper survey | n=NR, 15 metropolitan areas in 2014, 30 metropolitan areas in 2017  NR | n=NR, 15 metropolitan areas in 2014, 30 metropolitan areas in 2017  NR | VA vs. non-VA, 2014:  Primary care: ns; Dermatology: ns;  Cardiology: ns; Orthopedics: M 9.9d SD 4.7d vs. M 23.9d SD 8.1d, p<.001;  Overall: ns;  VA vs. non-VA, 2017:  Primary care: M 20.0d SD 10.4d vs. M 40.7d SD 35.0d, p=0.005;  Dermatology: M 15.6 d SD 12.2d vs. M 32.6d SD 16.5d, p<0.001;  Cardiology: M 15.3d SD 12.6d vs. M 22.8d SD 10.1d, p=0.04; Orthopedics: M 20.9d SD 13.3d vs. M 12.4d SD 5.5d, p=0.01;  Overall: ns | Statistics: Linear regression  Other methods of controlling: NR  Covariates: Metropolitan area, specialty | Y |  |
| Makarov, 2018^26^  Y (**National**)  Retrospective  Cancer  Clinical quality/safety  Cost/efficiency | 2004-2008, vs. non-Veterans in non-VA care; CDW vs. SEER Medicare | n=27,811  Low-risk men:  Guideline-concordant care: 60.6%;  Any imaging: 45.9%;  High-risk men:  Guideline-concordant care: 68.7%;  Any imaging: 75.3% | n=56,671  Low-risk men:  Guideline-concordant care: 53.1%;  Any imaging: 52.5%;  High-risk men:  Guideline-concordant care: 66.8%;  Any imaging: 76.8% | No statistical comparisons reported | Statistics: NR  Other methods of controlling: NR  Covariates: NR | Y |  |
| Wang, 2019^27^  Y (**National**)  Retrospective  ESRD  Clinical quality/safety | 2008-2013, vs. Veterans in VA-paid community care; VA enrollment, inpatient, outpatient, and purchased care data vs. Medicare enrollment, claims, and USRDS data | n=1100;  Two-year mortality: 24.5% | n=18,215  Two-year mortality: 41.8% | VA vs. Medicare, two-year mortality: hazard ratio 0.84 95% CI 0.73 to 0.96 | Statistics: Cox proportional hazards model  Other methods of controlling: NR  Covariates: Age, race/ethnicity, sex, employment status, regional and urban residential status, calendar year of dialysis initiation, baseline eGFR at dialysis initiation, receipt of pre-ESRD nephrology care within or outside the VA in the 2 years before ESRD onset, incident dialysis modality, type of vascular access at time of dialysis initiation, history of renal transplant, cause of ESRD, 29 indicators of diagnosed physical health conditions and mental health comorbidity, body mass index, hospitalization and institutionalization in the year before dialysis initiation, hospice use in the 90 days before dialysis initiation, dialysis in the inpatient setting, insurance coverage, VA copayment exempt status, distance to nearest VA outpatient dialysis unit and VAMC, degree of VA reliance for other outpatient care, presence of dialysis unit or nephrology services in nearest or most used VAMC, and FY11 occupancy rate of nearest VA outpatient dialysis unit. | Y |  |
| Thorpe, 2018^28^  Y (**National**)  Retrospective  Dementia  Clinical quality/safety | 2007-2010, Veterans in non-VA care; VA Medical SAS and VA PBM vs. Medicare MedPAR, Part D, and MBSF | n=35,647  Medication undersupply with no oversupply: 40%;  Medication oversupply with no undersupply: 9%;  Simultaneous medication oversupply and undersupply: 4% | n=9922  Medication undersupply with no oversupply: 47%;  Medication oversupply with no undersupply: 5%;  Simultaneous medication oversupply and undersupply: 3% | Non-VA vs. VA, odds ratio:  Medication undersupply with no oversupply: 1.13 95% CI 1.03 to 1.25;  Medication oversupply with no undersupply: 0.39 95% 0.32 to 0.47;  Simultaneous medication oversupply and undersupply: 0.48 95% CI 0.40 to 0.57 | Statistics: Multinomial logistic regression  Other methods of controlling: NR  Covariates: Age, sex, race/ethnicity, VA priority status, Medicaid status, distance to nearest VAMC, Elixhauser Comorbidity Index, use of memantine, number of VA ED and inpatient stays and use of VA home-based primary care in 2009, days alive in 2010, number of unique generic medications in 2010, and VISN indicator | Y |  |
| Vercammen-Grandjean, 2018^29^  Y (**National**)  Retrospective  COPD  Clinical quality/safety | 2007-2011, vs. non-Veterans in non-VA care; CDW vs. Medicare inpatient files | n=32,856  Participation in pulmonary rehabilitation after hospital discharge: n=485 | n=158,137  Participation in pulmonary rehabilitation after hospital discharge: n=3199 | VA vs. non-VA;  Participation in pulmonary rehabilitation after hospital discharge: 1.5% vs. 2% | Statistics: None  Other methods of controlling: NR  Covariates: NR | Y | No formal statistical comparison between VA and non-VA but sample size is large enough to estimate a significant difference |
| Wang, 2018^30^  Y (**National**)  Retrospective  Dialysis patients  Clinical quality/safety  Cost/efficiency | 2006-2013, vs. Veterans in VA-paid community care; VA Enrollment, MiniVitals, Patient Treatment, Outpatient Care, Fee Basis files vs. Medicare Beneficiary Summary, MedPAR, Outpatient, and Carrier files, and USRDS data | n=1101  Number of hospital days over 2 years follow-up period from chronic dialysis initiation: M 24.1 SD (37.2) | n=3085 (VA Purchase Care)  n=18,267 (Medicare)  Number of hospital days over 2 years follow-up period from chronic dialysis initiation:  VA-PC: M 22.4 SD (29.3);  Medicare: M 21.9 SD (26.0) | Number of hospital days over 2 years follow-up period from chronic dialysis initiation: VA vs. VA-PC,  incident rate ratio 0.97 95% CI 0.91 to 1.03,p=0.34;  vs. Medicare, incident rate ratio 0.98 95% CI 0.90 to 1.07,p=0.73;  VA vs. VA-PC or Medicare:  Risk of hospitalization after dialysis: p<0.0001, but authors note differences are not clinically meaningful;  Days of hospitalization after dialysis: p=0.80 | Statistics: Zero inflated negative binomial regression model  Other methods of controlling: NR  Covariates: Sex, urban vs. non-urban residence, year of chronic dialysis start date, employment status, factors surrounding dialysis initiation that would influence treatment setting (e.g., pre-ESRD nephrology care within or outside the VA, incident dialysis modality, type of vascular access at time of dialysis initiation, history of prior kidney transplant, cause of ESRD), distance to the nearest VA medical center (VAMC, i.e., the center most frequently used for non-dialysis care, else the nearest VAMC to residence), the extent of VA reliance for other outpatient care, initiated dialysis in the inpatient vs. outpatient setting, 29 indica- tors of diagnosed physical health conditions, BMI, hospitalization and institutionalization in the prior year, hospice use in the past 90 days, whether nearest VAMC had an on-site nephrology services or dialysis unit, and the 2011 fiscal year occupancy rate for nearest VAMC facility | Y | Outcomes not significantly different between healthcare systems |
| Augustine, 2018^6^  Y (**Regional**)  Retrospective  Kidney transplants  Access  Clinical quality/safety  Access | 2004-2016, non-Veterans in non-VA care; SRTR (VA and non-VA data) | n=3663  Median distance to transplant center: 282 miles | n=297,794  Median distance to transplant center: 22 miles | All kidney transplants:  VA vs. non-VA: adjusted hazard ratio (AHR) 0.72, 95% CI 0.68 to 0.76;  VA vs. Medicare: AHR 0.85, 95% CI 0.81 to 0.90;  VA vs. Medicaid: AHR 1.00, 95% CI 0.94 to 1.06;  Deceased donor kidney transplant:  VA vs. non-VA: AHR 0.85, 95% CI 0.80 to 0.90;  VA vs. Medicare: AHR 0.91, 95% CI 0.85 to 0.96;  VA vs. Medicaid: AHR 1.01, 95% CI 0.95 to 1.08;  Live donor kidney transplant:  VA vs. non-VA: AHR 0.51, 95% CI 0.46 to 0.57;  VA vs. Medicare: AHR 0.77, 95% CI 0.69 to 0.86;  VA vs. Medicaid: AHR 1.00, 95% CI 0.89 to 1.12;  Patient death:  VA vs. non-VA: AHR 0.89, 95% CI 0.82 to 0.97;  VA vs. Medicare: AHR 0.77, 95% CI 0.71 to 0.84;  VA vs. Medicaid: AHR 0.76, 95% CI 0.70 to 0.83;  Delisting from kidney transplant list due to "health deterioration" or "other":  VA vs. non-VA: AHR 1.38, 95% CI 1.26 to 1.51;  VA vs. Medicare: AHR, 1.1 95% CI 1.001 to 1.2;  VA vs. Medicaid: AHR 1.04, 95% CI 0.95 to 1.05 | Statistics: Cox models  Other methods of controlling: Matching VA to local non-VA facility  Covariates: Age group, race, gender, diagnosis group, time on dialysis at listing, candidate status  at listing, panel reactive antibody, BMI group, education, malignancy, peripheral vascular disease, region, year of listing, log  distance to center and community risk score | N | Note: * = p<0.05; RoB criteria not met: unbalanced samples |
| Anhang Price, 2018^31^  Y (**National**)  Retrospective  Inpatient and outpatient care  Clinical quality/safety Patient experience | 2014, vs. non-Veterans in non-VA care; CMS Hospital Compare (VA), VA Inpatient Evaluation Center, and VA Office of Performance Measurement vs. CMS Hospital Compare (non-VA) | N=135 facilities  In-hospital deaths per 1000 surgical discharges with serious treatable complications (inpatient): 100.6;  Postoperative pulmonary embolism or deep vein thrombosis rate (inpatient): 3.3;  Acute myocardial infarction 30-day readmission rate (inpatient): 18.6;  Heart failure 30-day mortality rate (inpatient): 11;  Evaluation of left ventricular systolic (LVS) function (inpatient): 99.8;  Prophylactic antibiotic received within 1 h prior to surgical incision (inpatient): 96.3;  Communication with doctors (inpatient): 77.1;  Care transition (inpatient): 53.7;  Overall rating of hospital (inpatient): 67.1;  Diabetes: Eye examination: 95.9%;  Tobacco use: advising smokers and tobacco users to quit (outpatient): 90.0%;  Hypertension: Controlling high blood pressure (diagnosis of hypertension, 18–85 years and < 140/90 mmHg): 76.1% | N=402 facilities  In-hospital deaths per 1000 surgical discharges with serious treatable complications (inpatient): 118.8;  Postoperative pulmonary embolism or deep vein thrombosis rate (inpatient): 4.6;  Acute myocardial infarction 30-day readmission rate (inpatient): 17.8;  Heart failure 30-day mortality rate (inpatient): 11.8;  Evaluation of left ventricular systolic (LVS) function (inpatient): 98.5;  Prophylactic antibiotic received within 1 h prior to surgical incision (inpatient): 98.5;  Communication with doctors (inpatient): 80.3;  Care transition (inpatient): 43.3;  Overall rating of hospital (inpatient): 70.3;  Diabetes: Eye examination: 84.6%  Tobacco use: advising smokers and tobacco users to quit (outpatient): 68.5%;  Hypertension: Controlling high blood pressure (diagnosis of hypertension, 18–85 years and < 140/90 mmHg): 65.5% | All VA and non-VA differences significant (p<0.05); last 3 comparisons: VA vs. Medicare HMO | Statistics: T-tests  Other methods of controlling: Matching VA to local non-VA facility  Covariates: Bed size (< 100 beds, 100–199 beds, and 200+ beds), Census division (East North Central, East South Central, Mid-Atlantic, Mountain, New England, Other, Pacific, South Atlantic, West North Central, and West South Central), location (urban, rural), and teaching status (teaching facility, nonteaching fa- cility) | Y |  |
| Kurella Tamura, 2018^32^  Y (**National**)  Retrospective  Pre-ESRD nephrology care  Clinical quality/safety | 2008-2011, vs. Veterans in non-VA care; VA administrative data vs. Medicare Claims, USRDS (both) | n=2966  Dialysis treatment within 2 years of incident kidney failure in pre-ESRD patients: 50.9% | n=2966  Dialysis treatment within 2 years of incident kidney failure in pre-ESRD patients: 79.2% | Medicare vs. VA  Dialysis treatment within 2 years of incident kidney failure in pre-ESRD patients: relative risk 1.56 95%, CI 1.50 to 1.62;  Mortality after receiving dialysis care for pre-ESRD patients: -8%, 95% CI -5% to -11%; | Statistics: Poisson regression; marginal standardization  Other methods of controlling: Propensity score matching  Covariates: Age, sex, race, marital status, VA co-pay, distance to nearest VA with nephrology services, Charlson Comorbidity Index, and rate of eGFR decline prior to incident kidney failure | Y |  |
| Barnett, 2018^33^  Y (**National**)  Retrospective  Elective coronary revascularization patients (PCI & CABG)  Clinical quality/safety Access  Cost/efficiency | 2008-2011, vs. Veterans in VA-paid community care; VA and non-VA: ArcGIS, VA Vital Status File, VA Managerial Cost Accounting System | n=15,340  Total cost (procedure + readmission + travel), PCI: M $15,683.00 SD ($16,493.00);  Total cost (procedure + readmission + travel), CABG: M $63,144.00 SD ($46,018.00);  Actual distance traveled, PCI: M 90.8  Actual distance traveled, CABG: M 123.2 | n=3715  Total cost (procedure + readmission + travel), PCI: M $22,025.00  SD ($30,701.00);  Total cost (procedure + readmission + travel), CABG: M $55,526.00 SD ($74,797.00);  Actual distance traveled, PCI: M 60.1  Actual distance traveled, CABG: M 81.5 | 30-day mortality, PCI: VA>non-VA, relative risk (RR) 2.40 95% CI 1.57 to 3.66, p<0.001;  30-day mortality, CABG: VA=non-VA, RR 0.89 95% CI 0.45 to 1.77, p=0.74;  30-day readmissions, PCI: VA=non-VA, RR 0.96 95% CI 0.79 to 1.16, p=0.68;  30-day readmissions, CABG: VA=non-VA, RR 1.16 95% CI 0.89 to 1.50, p=0.28;  Total cost (procedure + readmission + travel), PCI: VA<non-VA, p<0.001;  Total cost (procedure + readmission + travel), CABG: VA>non-VA, p<0.001;  Actual distance traveled, PCI: VA>non-VA, p<0.001;  Actual distance traveled, CABG: VA>non-VA, p=0.002 | Statistics: Generalized estimating equations  Other methods of controlling: Propensity weighting  Covariates: age, sex, race/ethnicity, recent myocardial infarction, prior PCI, prior CABG surgery, cerebrovascular disease, peripheral vascular disease, congestive heart failure, type 1 and type 2 diabetes, body mass index, renal function, dialysis, chronic obstructive pulmonary disease, atrial fibrillation, and the number of vessels revascularized | Y |  |
| Heidenrich, 2017^11^  Y (**National**)  Retrospective  Hospital care  Patient experience | 2014; vs. non-Veterans in non-VA care; Yelp (both) | n=39 facilities  Patient ratings (weighted for number of reviews): M 3.70 SD 0.74 | n=39 facilities  Patient ratings (weighted for number of reviews): M 3.19 SD 0.54 | VA vs. non-VA:  Difference in ratings, weighted by review count: p=0.0025  Covariate adjusted rating difference 0.65, 95% CI 0.18 to 1.12 | Statistics: Multivariate regression  Other methods of controlling: Local affiliate matching  Covariates: Bed size, membership in COTH, pres- ence of an accredited graduate medical education program, and certification by TJC | N | RoB criteria not met: analysis of Yelp reviews of only 39 of 131 VA facilities due to lack of data |
| Blay, 2017^34^  Y (**National**)  Retrospective  Hospital care  Clinical quality/safety  Patient experience | 2012-2015, vs. non-Veterans in non-VA care; Both VA and non-VA: Hospital Compare, AHA Annual Survey | n=129 facilities Pressure ulcers: M 0.28, 95% CI 0.21 to 0.27;  Death among surgical inpatients with serious treatable conditions: M 105.82, 95% CI 96.7 to 114.92;  Iatrogenic pneumothorax: M 0.27, 95% CI 0.22 to 0.32;  30-day mortality, AMI: 9.27, 95% CI 9.0 to 9.46;  30-day readmissions, AMI: M 15.59 95% CI, 15.45 to 15.74;  Doctor communication: top box 76.70%, 95% CI 76.01 to 77.39%;  Cleanliness: top box 73.41% 95% CI 71.95 to 74.87%;  Care transition: top box 53.62%, 95% CI 51.79% to 54.46%;  Quietness: 55.80% , 95% CI 54.24% to 57.37%;  Recommendation of hospital to others: top box, 67.92% 95% CI 66.56 to 69.28%; | n=4010 facilities  Pressure ulcers: M 0.44, 95% CI 0.44 to 0.46;  Death among surgical inpatients with serious treatable conditions: M 136.34, 95% CI 135.42 to 137.26;  Iatrogenic pneumothorax: M 0.41, 95% CI 0.40 to 0.41;  30-day mortality, AMI: M 14.1, 95% CI 14.04 to 14.15;  30-day readmissions, AMI: M 16.89, 95% CI 16.84 to 16.94;  Doctor communication:  top box 82.14%, 95% CI 81.95 to 82.32%;  Cleanliness: 74.14%, 95% CI 73.86% to 74.41%;  Care transition: top box 52.71%, 95% CI 52.47% to 52.96%;  Quietness: top box 62.93 %, 95% CI 62.59% to 63.26%;  Recommendation of hospital to others: top box 71.66%, 95% CI 71.33% to, 71.99%; | VA<non-VA for all clinical quality/safety outcomes, p<0.03;  Non-VA>VA for all patient experience outcomes (p<0.005) except cleanliness and care transition | Statistics: T-tests  Other methods of controlling: Outcomes were rates per 1000 discharges; Bonferroni correction  Covariates: NR | Y |  |
| Mody, 2017^12^  N (**NA**)  Prospective survey  Nursing home care  Clinical quality/safety | 2014-2015; vs. non-Veterans in non-VA care; Original surveys (both VA and non-VA data) | n=47 facilities  Policy for appropriate indications for catheter use: 63.8%;  Policy for urinary catheter maintenance: 78.7%;  Urinary catheters removed within 24–48 hrs. of admission unless there are appropriate: 74.5%;  Catheter-associated urinary tract infection surveillance performed: 93.6% | n=306 facilities  Policy for appropriate indications for catheter use: 81.4%;  Policy for urinary catheter maintenance: 92.8%;  Urinary catheters removed within 24–48 hrs. of admission unless there are appropriate: 93.8%;  Catheter-associated urinary tract infection surveillance performed: 65.7% | Policy for appropriate indications for catheter use: VA<non-VA, p=0.004;  Policy for urinary catheter maintenance: VA<non-VA, p=0.001;  Urinary catheters removed within 24–48 hrs. of admission unless there are appropriate: VA<non-VA, p<0.001;  Catheter-associated urinary tract infection surveillance performed: VA>non-VA, p<0.001 | Statistics: Multivariable logistic regression models  Other methods of controlling: All nursing homes participating in AHRQ HAI/CAUTI patient safety collaborative  Covariates: Number of residents in facility, short-term sub-acute rehabilitation offered, presence of an HAI committee, infection prevention training, and infection preventionist with 3 or more years of experience | N | RoB criteria not met: data from only half of states |
| Shields, 2017^14^  Y (**National**)  Retrospective  Psychiatric care  Clinical quality/safety | 2014, vs. non-Veterans in non-VA care; HBIPS | n=105 facilities  NR | n=141 facilities (for-profit), 180 (non-VA government)  NR | For-profit vs. VA:  Admissions screening for inpatient psychiatric care: 37.2%, p<0.001;  Restraint hours per 1000 patient hours: -77.9%, p=0.004;  Seclusion hours per 1000 patient hours: -61.6%, p=0.01;  Creating a continuing care plan at discharge: 41.7%, p<0.001;  Transmitting a continuing care plan at discharge: 40.4%, p<0.001;  Non-VA government vs. VA:  Appropriate justification of antipsychotics at discharge: 33.9%, p<0.001 | Statistics: T-tests  Other methods of controlling: NR  Covariates: NR | N | RoB criteria not met: no adjustment for patient characteristics |
| Burke, 2016^7^  Y (**National**)  Retrospective  Headache and neuropathy  Clinical quality/safety | 2004-2011, vs. non-Veterans in non-VA care; CDW vs. MedPAR/HRS | n=256,608  Imaging for nontraumatic headache: 22.1%;  Imaging for nontraumatic headache excluding cancer, hemiplegic migraine, giant cell arteritis, epilepsy, cerebrovascular disease including TIA, head or neck trauma, altered mental status, personal history of stroke/TIA or cancer, multiple sclerosis, or dementia: 15.3%;  Imaging for migraine excluding cancer, hemiplegic migraine, giant cell arteritis, epilepsy, cerebrovascular disease including TIA, head or neck trauma, altered mental status, personal history of stroke/TIA or cancer, multiple sclerosis, or dementia: 7.1%;  Neuroimaging any component of neuroaxis: 9%;  Neuroimaging any component of neuroaxis excluding cancer, hemiplegic migraine, giant cell arteritis, epilepsy, cerebrovascular disease including TIA, head or neck trauma, altered mental status, personal history of stroke/TIA or cancer, multiple sclerosis, or dementia: 6.1% | n=2005  Imaging for nontraumatic headache: 49.0%;  Imaging for nontraumatic headache excluding cancer, hemiplegic migraine, giant cell arteritis, epilepsy, cerebrovascular disease including TIA, head or neck trauma, altered mental status, personal history of stroke/TIA or cancer, multiple sclerosis, or dementia: 27.1%;  Imaging for migraine excluding cancer, hemiplegic migraine, giant cell arteritis, epilepsy, cerebrovascular disease including TIA, head or neck trauma, altered mental status, personal history of stroke/TIA or cancer, multiple sclerosis, or dementia: 15.6%;  Neuroimaging any component of neuroaxis: 23.7%;  Neuroimaging any component of neuroaxis excluding cancer, hemiplegic migraine, giant cell arteritis, epilepsy, cerebrovascular disease including TIA, head or neck trauma, altered mental status, personal history of stroke/TIA or cancer, multiple sclerosis, or dementia: 15% | VA<non-VA for all outcomes, p<0.001; except for imaging for migraine, p=0.027 | Statistics: T-tests  Other methods of controlling: NR  Covariates: NR | N | RoB criteria not met: unbalanced samples |
| Lee, 2017^8^  Y (**National**)  Retrospective  Headache and neuropathy  Access | 2010-2011, vs. non-Veterans in non-VA care; Both VA and non-VA: Health Tracking Household Survey | n=203  Self-reported delay in care in last 12 months: M 28.68%, 95% CI 20.18% to 39.0% | n=10,719  Self-reported delay in care in last 12 months:  Commercial: M 17.3, 95% CI 16.18% to 18.49%;  Medicare: M 17.97 %, 95% CI 13.88% to 22.87%;  Medicaid/other: M 15.26%, 95% CI 12.55% to 18.43% | Self-reported delay in care in last 12 months: VA vs. commercial: adjusted odds ratio 1.76, 95% CI 1.11 to 2.80, p<0.05 | Statistics: Multivariate logistic regression  Other methods of controlling: NR  Covariates: Perceived general health status, perceived health care satisfaction, age, gender, education, annual family income, race, and region | N | RoB criteria not met: unbalanced samples |
| Axon, 2016^13^  Y (**Regional**)  Retrospective  Heart failure  Clinical quality/safety | 2007-2011, vs. Veterans in non-VA care; CDW vs. Medicare inpatient, outpatient, and carrier files | n=2242  Emergency department visits:  All cause: M 72.6 SD (79.0);  HF-related: M 6.2 SD (22.8);  Hospitalizations:  All cause: M 31.5 SD (56.7);  HF-related: M 6.5 SD (27.1);  30-day readmissions:  All cause: M 30.6 SD (54.6);  HF-related: M 6.4 SD (27.0) | n=8825  Emergency department visits: All cause: M 45.0 SD (67.5);  HF-related: M 3.6 SD (12.6);  Hospitalizations:  All cause: M 26.0 SD (34.5);  HF-related: M 2.8 SD (12.4);  30-day readmissions:  All cause: M 23.2 SD (32.4);  HF-related: M 2.2 SD (10.2) | Non-VA vs. VA  Emergency department visits: All cause: adjusted odds ratio (AOR) 0.62, 95% CI 0.60 to 0.64;  HF-related: AOR 0.60, 95% CI 0.55 to 0.66;  Hospitalizations:  All cause: AOR 0.98, 95% CI 0.95 to 1.02;  HF-related: AOR 0.61, 95% CI 0.55 to 0.68;  30-day readmissions:  All cause: AOR 0.87, 95% CI 0.83 to 0.90;  HF-related: AOR 0.51, 95% CI 0.46 to 0.57 | Statistics: Zero-inflated negative binomial models  Other methods of controlling: NR Covariates: Age, race, gender, year of visit, dual use category, year of visit, and comorbidities that were found to be significant using a stepwise selection procedure | N | P-values not reported; RoB criteria not met: data only from South Carolina |
| Jia, 2016^35^  Y (**National**)  Retrospective  Nursing home care  Clinical quality/safety | 2006-2009, vs. Veterans in VA-paid community care; VA MDS 2.0 vs. CMS MDS 2.0 | n=12,660  Rehabilitation therapy: 75.5%;  Restorative nursing care: 33.% | n=5612  Rehabilitation therapy: 76.4%;  Restorative nursing care: 30.6% | VA vs. non-VA:  Rehabilitation therapy: adjusted odds ratio (AOR) 1.16, 95% CI 1.01 to 1.32, p=0.033;  Restorative nursing care: AOR 2.28, 95% CI 2.02 to 2.57, p<0.0001 | Statistics: 2-part log-linear model  Other methods of controlling: NR Covariates: Gender, education, depression score, ADL score, cognition score, comorbidity index score, number of assessments, facility region, facility rurality, facility hospital status, facility beds, facility resident-to-bed ratio | Y |  |
| Watkins, 2016^36^  Y (**National**)  Retrospective  Schizophrenia, bipolar disorder, posttraumatic stress disorder, major depression, and substance use disorders  Clinical quality/safety | FY07-FY08, vs. non-Veterans in non-VA care; VA inpatient, laboratory and pharmacy files vs. Thomson-Reuters MarketScan Commercial Claims and Encounter Database | n=836,519  Medication laboratory tests: 77.4%;  Any laboratory screening tests: 86.9%;  Antipsychotics, 12-week supply: 50.0%;  Maintenance antipsychotics: 37.4%;  Maintenance mood stabilizers: 31.3%;  Antidepressants, 12-week supply: 49.0%;  Maintenance antidepressants: 31.3% | n=545,484  Medication laboratory tests: 5.8%;  Any laboratory screening tests: 49.7%;  Antipsychotics, 12-week supply: 22.8%;;  Maintenance antipsychotics: 23.1%  Maintenance mood stabilizers: 20.3%;;  Antidepressants, 12-week supply: 20.2%  Maintenance antidepressants: 13.1% | VA>non-VA for all outcomes, p<0.001 | Statistics: NR  Other methods of controlling: NR Covariates: Age, gender | Y |  |
| Jones, 2015^4^  N (**NA**)  Retrospective analysis of RCT  Advanced chronic systolic heart failure  Clinical quality/safety | 1999, vs. non-Veterans in non-VA care; BEST data (VA and non-VA) | n=898  NR | n=1216 | VA vs. non-VA:  All-cause mortality among patients with advanced chronic systolic HF: adjusted odds ratio (AOR) 0.94, 95% CI 0.80 to 1.10, p=0.448;  Cardiovascular mortality among patients with advanced chronic systolic HF: AOR 0.92, 95% CI 0.74 to 1.10, p=0.359;  HF mortality among patients with advanced chronic systolic HF: AOR 0.76, 95% CI 0.57 to 1.02, p=0.064;  Sudden cardiac death among patients with advanced chronic systolic HF: AOR 1.05, 95% CI 0.83 to 1.03, p=0.664;  Mortality due to AMI among patients with advanced chronic systolic HF: AOR 3.12, 95% CI 1.19 to 8.19, p=0.021;  All-cause hospitalization among patients with advanced chronic systolic HF: AOR 0.99 95%, CI 0.88 to 1.10; p=0.868;  HF hospitalization among patients with advanced chronic systolic HF: AOR 0.88, 95% CI 0.76 to 1.02, p=0.092 | Statistics: Cox proportional hazard models  Other methods of controlling: NR Covariates: Age, race, body mass index, smoking, HF duration, coronary artery disease, diabetes mellitus, hypertension, atrial fibrillation, peripheral vascular disease, chronic kidney disease, randomization to bucindolol, use of angiotensin-converting enzyme inhibitors or angiotensin- receptor blockers, digoxin, and diuretics, NYHA class symptoms, LVEF and right ventricular EF (RVEF), cardiothoracic ratio, pulmonary edema, heart rate, systolic and diastolic blood pressure, hemoglobin, serum creatinine, and serum cholesterol | N | RoB criteria not met: clinical trial sample |
| Chan, 2022^37^  Y (**National**)  Retrospective  Emergency department care  Clinical quality/safety | 2001-2018, vs. Veterans in non-VA care; CDW and VBA death records vs. Medicare claims and SSA death records | n=231,611  30-day mortality after ambulance ride: 9.32 deaths per 100 patients, 95% CI 9.15 to 9.50 | n=1,238,546  30-day mortality after ambulance ride: 11.67 deaths per 100 patients, 95% CI 11.58 to 11.76 | VA vs. non-VA  30-day mortality after ambulance ride: difference  -2.35 deaths per 100 patients, 95% CI -2.16 to -2.54 | Statistics: Ordinary least squares regression  Other methods of controlling: NR Covariates: Zip code of residence, demographic characteristics (age in two year bands, race or ethnic origin, and sex), six binary variables indicating receipt of VA or non-VA primary care, emergency care, and inpatient care in the 12 months before the ride, and previous medical diagnoses, specified as 31 indicators for Elixhauser comorbidities recorded in the 12 months before the ride, origin of the ride (residence; residential, domiciliary, or custodial facility; skilled nursing facility; or scene of accident or acute event), time (day of the week, month-year interactions), life support capabilities, classified according to categories for basic and advanced life support specified in the Healthcare Common Procedure Coding System codes, and primary diagnosis made during the ride, coded according to ICD-9 | Y |  |
| Florez, 2021^5^  N (**NA**)  Retrospective analysis of RCT  Type 2 diabetes  Clinical quality/safety | NR, vs. non-Veterans in non-VA care; GRADE data (both VA and non-VA) | n=1216  HbA1c < 7% (<53 mmol/mol) among patients with a history of CVD: 18.1%  BP < 140/90 mmHg among patients with a history of CVD: 80.2%;  Treated for HTN among patients with a history of CVD: 93.7%;  LDLc < 70 mg/dL (1.8 mmol/L) among patients with a history of CVD: 50.0%;  LDLc < 100 mg/dL (2.6 mmol/L) among patients with a history of CVD: 81.1%;  Statin use among patients with a history of CVD: 87.4%;  Aspirin use among patients with a history of CVD: 81.9%;  HbA1c < 7% (<53 mmol/mol) among patients with a history of CVD: 15.1%;  BP < 140/90 mmHg among patients with no history of CVD: 73.6%;  Treated for HTN among patients with no history of CVD: 74.9%;  LDLc < 70 mg/dL (1.8 mmol/L) among patients with no history of CVD: 34.9%;  LDLc < 100 mg/dL (2.6 mmol/L) among patients with a history of CVD: 68.2%;  Statin use among patients with no history of CVD:  Aspirin use among patients with no history of CVD: 70.8%;  HbA1c < 7% (<53 mmol/mol) among patients with no history of CVD: 46.6% | n=3831  HbA1c < 7% (<53 mmol/mol) among patients with a history of CVD: 10.9%  BP < 140/90 mmHg among patients with a history of CVD: 70.1%;  Treated for HTN among patients with a history of CVD: 93.0%;  LDLc < 70 mg/dL (1.8 mmol/L) among patients with a history of CVD: 36.9%;  LDLc < 100 mg/dL (2.6 mmol/L) among patients with a history of CVD: 74.4%;  Statin use among patients with a history of CVD: 84.1%;  Aspirin use among patients with a history of CVD: 76.6%;  HbA1c < 7% (<53 mmol/mol) among patients with a history of CVD: 14.2%;  BP < 140/90 mmHg among patients with no history of CVD: 76.0%;  Treated for HTN among patients with no history of CVD: 65.4%;  LDLc < 70 mg/dL (1.8 mmol/L) among patients with no history of CVD: 24.2%;  LDLc < 100 mg/dL (2.6 mmol/L) among patients with a history of CVD: 62.9% ;  Statin use among patients with no history of CVD:  Aspirin use among patients with no history of CVD: 59.5%;  HbA1c < 7% (<53 mmol/mol) among patients with no history of CVD: 40.2% | VA>non-VA, adjusted analyses  BP < 140/90 mmHg among patients with a history of CVD: p=0.035  Treated for HTN among patients with no history of CVD: p=0.006  LDLc < 70 mg/dL (1.8 mmol/L) among patients with no history of CVD: p=0.045  Aspirin use among patients with no history of CVD: p=0.028  HbA1c < 7% (<53 mmol/mol) among patients with no history of CVD: p=0.003 | Statistics: Pearson’s chi- squared test with Yates’ continuity correction  Other methods of controlling: NR Covariates: Age, sex, race, and ethnicity | N | RoB criteria not met: clinical trial sample |
| Feyman, 2022^38^  Y (**National**)  Retrospective  Primary, specialty, and mental health care  Access | 2018-2021, vs. Veterans in VA-paid community care; CDW (VA and non-VA) | n=4,016,156  Average wait times:  Primary care: 29.0 (SD 5.5) days;  Mental health care: 33.6 (SD 4.6) days;  All other specialties: 35.4 (SD 2.7) days | n=3,042,060  Average wait times:  Primary care: 38.9 (SD 8.2) days;  Mental health care: 43.9 (SD 9.0) days;  All other specialties: 41.9 (SD 5.9) days | Average wait times:  Primary care: VA<non-VA in 15 of 18 VISNs;  Mental health:  VA<non-VA in 16 of 18 VISNs;  All other specialties: VA<non-VA in 17 of 18 VISNs | Statistics: Ordinary least squares regressions; 2-sided t-tests Other methods of controlling: NR  Covariates: Specialty mix (distribution of stop codes), VISN | Y |  |
| Presley, 2022^15^  Y (**National**)  Retrospective  Nonsmall lung cancer  Clinical quality/safety | 2006-2012, vs. non-Veterans in non-VA care; Veterans Central Cancer Registry (VACCR) vs. Surveillance, Epidemiology, and End Results (SEER) and Medicare claims | n=18,054  Change in aggressive care at end of life between 2006 and 2012:  -15.0% (46.0% to 31.0%) | n=13,277  Change in aggressive care at end of life between 2006 and 2012:  -3.8% (41.9% to 38.0%) | Change in aggressive care at end of life between 2006 and 2012: VA>non-VA, p<0.001;  % change in hospice admissions in Medicare hospital referral region on aggressive care at matched VA facility: AOR 0.13, 95% CI 0.08 to 0.23 | Statistics: Chi-square tests  Other methods of controlling: NR  Covariates: Age, sex, race, comorbidities | N | No adjustment for demographic covariates in main analysis; composition of multicomponent aggressive care measure unclear |
| Pickering, 2022^39^  Y (**National**)  Retrospective  Low-value prostate-specific antigen (PSA) testing  Cost/efficiency | FY2017-FY2018, vs. Veterans in non-VA care; CDW, Area Resource File, and VHA Service Support Center vs. Beneficiary Summary File, Medicare Provider Analysis and Review, Inpatient, Skilled Nursing Facility, Outpatient, Home Health Agency, Hospice, Durable Medical Equipment, and Carrier  files | n=36,469  Total downstream or “cascade” services related to low-value PSA testing: 53.9 services/100 Veterans;  Cost of cascade services related to low-value PSA testing: $45.1/Veteran | n=17,981  Total downstream or “cascade” services related to low-value PSA testing: 45.3 services/100 Veterans;  Cost of cascade services related to low-value PSA testing: $35.0/Veteran | Non-VA vs. VA  Adjusted difference in downstream or “cascade” services related to low-value PSA testing: 9.9 services/100 Veterans, 95% CI 9.7 to 10.1;  Adjusted cost of cascade services related to low-value PSA testing: $11.9/Veteran, 95% CI $7.6 to $16.2 | Statistics: Negative binomial models; weighted linear regression  Other methods of controlling: Stabilized inverse probability of treatment weights  Covariates: Age, race and ethnicity, VA priority group, driving distance to the nearest VA facility, number of Elixhauser conditions, individual Elixhauser conditions, academic affiliation, facility size, census region, rurality, and complexity level at the VA medical center-level | Y |  |
| Fortney, 2022^9^  N (**NA**)  Prospective survey  In-person- and tele-mental health care  Access  Cost/efficiency  Patient experience  Clinical quality/safety | 2019-2020, vs. Veterans in VA-paid community care; CDW and telephone survey | n=303  Number of barriers to care: M 0.9, SD 1.3; Number of encounters: M 5.9, SD 7.3;  Patient centeredness:  M 4.3, SD 0.6;  Change in PHQ-8 (depression symptoms): M  -1.2, SD -4.9;  Change in PCL-5 (post-traumatic stress disorder symptoms): M  -3.4, SD -12.5 | n=242  Number of barriers to care: M 1.3, SD 1.6;  Number of encounters: M 6.2, SD 6.8;  Patient centeredness: M 4.2, SD 0.7;  Change in PHQ-8 (depression symptoms): M  -2.2, SD -5.3;  Change in PCL-5 (post-traumatic stress disorder symptoms): M  -6.0, SD -12.6 | Number of access-related barriers to care: VA<non-VA: p<0.001;  Number of encounters: VA=non-VA; p=0.276;  Patient centeredness: VA=non-VA; p=0.243;  Change in PHQ-8 (depression symptoms): VA>non-VA; p=0.011;  Change in PCL-5 (post-traumatic stress disorder symptoms): VA=non-VA; p=0.148 | Statistics: Multivariate statistical analyses; chi-square and t-tests  Other methods of controlling: NR  Covariates: Provisional diagnosis, suicidality, rurality, and prior VA mental health use | N | Small sample size |
| Scheuner, 2022^40^  Y (**National**)  Retrospective  Genetic counseling  Clinical quality/safety | 2010-2017, vs. Veterans in VA-paid community care; CDW | n=6775  Genetic referrals completed (% of total referrals): 5073 (74.9%) | n=3423  Genetic referrals completed (% of total referrals): 1961 (57.3%) | Non-VA vs. VA:  Completed genetic consultations: OR 0.43, 95% CI 0.28 to 0.65;  Follow-up cancer surveillance and risk-reducing procedures among those who completed a genetic consultation: OR 0.64, 95% CI 0.52 to 0.78 | Statistics: Multivariate regression models  Other methods of controlling: NR  Covariates: Genetic referral models: care model x age, x race or ethnicity, and x gender interactions; Risk-reducing surveillance/ procedures models: care model x consultation status interactions, and cardiovascular disease | Y |  |
| Petros, 2022^10^  Y (**Local**)  Retrospective  Colorectal cancer  Clinical quality/safety  Access | 2015-2018, vs. Veterans in VA-paid, community care; Chart review | n=235  Adenomas detection (adenoma detection rate): 147 (62.6%);  Compliance with surveillance guidelines: 93.3%;  Time to colonoscopy: M 83.8 days, 95% CI 45.2 to 122.4 days | n=235  Adenomas detection (adenoma detection rate): 86 (36.7%);  Compliance with surveillance guidelines: 74.9%;  Time to colonoscopy: M 58.4 days, 95% CI 24.7 to 92.1 days | Non-VA vs. VA:  Adenoma detection rate: OR 0.39, 95% CI 0.25 to 0.63;  Compliance with surveillance guidelines: OR 0.21, 95% CI 0.09 to 0.45;  Time to colonoscopy: non-VA<VA, p<0.0001 | Statistics: Multivariate logistic regression  Other methods of controlling: NR  Covariates: (Adenoma model) Diabetes mellitus, preparation quality adequate, and cecal intubation; (Guideline model) adenoma detected, performed by non-gastroenterologist, screening indication, surveillance indication, and adequate bowel preparation | N | Small sample size; only one facility sample; no demographic controls in statistical models |

**Supplementary Material 6. Risk of bias table for included studies**

| **Author, Year** | **Time frames** | **Samples (both VA and non-VA)** | **How did the specifications for the outcome assessments compare in VA and non VA samples?** | **Statistical methods** |
| --- | --- | --- | --- | --- |
| Nuti, 2016^16^ | Contemporaneous time frames | Representative or national samples (both VA and non-VA) | Identical | Sufficient sample size and/or methods appropriate to address hypothesis(ses) |
| Vanneman, 2020^17^ | Contemporaneous time frames | Representative or national samples (both VA and non-VA) | Identical | Sufficient sample size and/or methods appropriate to address hypothesis(ses) |
| Gurewich, 2021^18^ | Contemporaneous time frames | Representative or national samples (both VA and non-VA) | Identical | Sufficient sample size and/or methods appropriate to address hypothesis(ses) |
| Davila, 2021^19^ | Contemporaneous time frames | Representative or national samples (both VA and non-VA) | Identical | Sufficient sample size and/or methods appropriate to address hypothesis(ses) |
| Intrator, 2021^20^ | Contemporaneous time frames | Representative or national samples (both VA and non-VA) | Identical | Sufficient sample size and/or methods appropriate to address hypothesis(ses) |
| LaBedz, 2021^21^ | Contemporaneous time frames | Representative or national samples (both VA and non-VA) | Identical | Sufficient sample size and/or methods appropriate to address hypothesis(ses) |
| Gidwani, 2021^22^ | Contemporaneous time frames | Representative or national samples (both VA and non-VA) | Identical | Sufficient sample size and/or methods appropriate to address hypothesis(ses) |
| Griffith, 2020^23^ | Contemporaneous time frames | Representative or national samples (both VA and non-VA) | Identical | Sufficient sample size and/or methods appropriate to address hypothesis(ses) |
| Gidwani-Marszowski, 2020^24^ | Contemporaneous time frames | Representative or national samples (both VA and non-VA) | Identical | Sufficient sample size and/or methods appropriate to address hypothesis(ses) |
| Penn, 2019^25^ | Contemporaneous time frames | Representative or national samples (both VA and non-VA) | Identical | Sufficient sample size and/or methods appropriate to address hypothesis(ses) |
| Makarov, 2018^26^ | Contemporaneous time frames | Representative or national samples (both VA and non-VA) | Identical | Sufficient sample size and/or methods appropriate to address hypothesis(ses) |
| Wang, 2019^27^ | Contemporaneous time frames | Representative or national samples (both VA and non-VA) | Identical | Sufficient sample size and/or methods appropriate to address hypothesis(ses) |
| Thorpe, 2018^28^ | Contemporaneous time frames | Representative or national samples (both VA and non-VA) | Identical | Sufficient sample size and/or methods appropriate to address hypothesis(ses) |
| Vercammen-Grandjean, 2018^29^ | Contemporaneous time frames | Representative or national samples (both VA and non-VA) | Identical | Sufficient sample size and/or methods appropriate to address hypothesis(ses) |
| Wang, 2018^30^ | Contemporaneous time frames | Representative or national samples (both VA and non-VA) | Identical | Sufficient sample size and/or methods appropriate to address hypothesis(ses) |
| Augustine, 2018^6^ | Contemporaneous time frames | All between A and C | Identical | Sufficient sample size and/or methods appropriate to address hypothesis(ses) |
| Anhang Price, 2018^31^ | Contemporaneous time frames | Representative or national samples (both VA and non-VA) | Identical | Sufficient sample size and/or methods appropriate to address hypothesis(ses) |
| Kurella Tamura, 2018^32^ | Contemporaneous time frames | Representative or national samples (both VA and non-VA) | Identical | Sufficient sample size and/or methods appropriate to address hypothesis(ses) |
| Barnett, 2018^33^ | Contemporaneous time frames | Representative or national samples (both VA and non-VA) | Identical | Sufficient sample size and/or methods appropriate to address hypothesis(ses) |
| Heidenreich, 2017^11^ | Contemporaneous time frames | All between A and C | Identical | All between A and C |
| Blay, 2017^34^ | Contemporaneous time frames | Representative or national samples (both VA and non-VA) | Identical | Sufficient sample size and/or methods appropriate to address hypothesis(ses) |
| Mody, 2017^12^ | Contemporaneous time frames | All between A and C | Identical | Sufficient sample size and/or methods appropriate to address hypothesis(ses) |
| Shields, 2017^14^ | Contemporaneous time frames | All between A and C | Identical | Sufficient sample size and/or methods appropriate to address hypothesis(ses) |
| Burke, 2016^7^ | Contemporaneous time frames | Small, limited, unequal or non-representative samples | Identical | Insufficient sample size and/or methods questionable to address hypothesis(ses) |
| Lee, 2017^8^ | Contemporaneous time frames | All between A and C | Identical | All between A and C |
| Axon, 2016^13^ | Contemporaneous time frames | All between A and C | Identical | All between A and C |
| Jia, 2016^35^ | Contemporaneous time frames | Representative or national samples (both VA and non-VA) | Identical | Sufficient sample size and/or methods appropriate to address hypothesis(ses) |
| Watkins, 2016^36^ | Contemporaneous time frames | Representative or national samples (both VA and non-VA) | Identical | Sufficient sample size and/or methods appropriate to address hypothesis(ses) |
| Jones, 2015^4^ | Contemporaneous time frames | All between A and C | Identical | Sufficient sample size and/or methods appropriate to address hypothesis(ses) |
| Chan, 2022^37^ | Contemporaneous time frames | Representative or national samples (both VA and non-VA) | Identical | Sufficient sample size and/or methods appropriate to address hypothesis(ses) |
| Florez, 2021^5^ | Contemporaneous time frames | All between A and C | Identical | Sufficient sample size and/or methods appropriate to address hypothesis(ses) |
| Feyman, 2022^38^ | Contemporaneous time frames | Representative or national samples (both VA and non-VA) | Identical | Sufficient sample size and/or methods appropriate to address hypothesis(ses) |
| Presley, 2022^15^ | Contemporaneous time frames | Representative or national samples (both VA and non-VA) | Identical | All between A and C |
| Pickering, 2022^39^ | Contemporaneous time frames | Representative or national samples (both VA and non-VA) | Identical | Sufficient sample size and/or methods appropriate to address hypothesis(ses) |
| Fortney, 2022^9^ | Contemporaneous time frames | All between A and C | Identical | Sufficient sample size and/or methods appropriate to address hypothesis(ses) |
| Scheuner, 2022^40^ | Contemporaneous time frames | Representative or national samples (both VA and non-VA) | Identical | Sufficient sample size and/or methods appropriate to address hypothesis(ses) |
| Petros, 2022^10^ | Contemporaneous time frames | All between A and C | Identical | All between A and C |

**Supplementary Material 7. Supplementary material references**

1. Institute of Medicine. *Crossing the Quality Chasm: A New Health System for the 21st Century*. National Academies Press; 2001.

2. Trivedi AN, Matula S, Miake-Lye I, Glassman PA, Shekelle P, Asch S. Systematic review: comparison of the quality of medical care in Veterans Affairs and non-Veterans Affairs settings. *Med Care*. Jan 2011;49(1):76-88. doi:10.1097/MLR.0b013e3181f53575

3. O'Hanlon C, Huang C, Sloss E, et al. Comparing VA and Non-VA Quality of Care: A Systematic Review. *J Gen Intern Med*. Jan 2017;32(1):105-121. doi:10.1007/s11606-016-3775-2

4. Jones LG, Sin MK, Hage FG, et al. Characteristics and outcomes of patients with advanced chronic systolic heart failure receiving care at the Veterans Affairs versus other hospitals: insights from the Beta-blocker Evaluation of Survival Trial (BEST). *Circ Heart Fail*. Jan 2015;8(1):17-24. doi:10.1161/circheartfailure.114.001300

5. Florez HJ, Ghosh A, Pop-Busui R, et al. Differences in complications, cardiovascular risk factor, and diabetes management among participants enrolled at veterans affairs (VA) and non-VA medical centers in the glycemia reduction approaches in diabetes: A comparative effectiveness study (GRADE). *Diabetes Res Clin Pract*. Dec 28 2021;184:109188. doi:10.1016/j.diabres.2021.109188

6. Augustine JJ, Arrigain S, Balabhadrapatruni K, Desai N, Schold JD. Significantly Lower Rates of Kidney Transplantation among Candidates Listed with the Veterans Administration: A National and Local Comparison. *J Am Soc Nephrol*. Oct 2018;29(10):2574-2582. doi:10.1681/asn.2017111204

7. Burke JF, Kerr EA, McCammon RJ, Holleman R, Langa KM, Callaghan BC. Neuroimaging overuse is more common in Medicare compared with the VA. *Neurology*. Aug 23 2016;87(8):792-8. doi:10.1212/wnl.0000000000002963

8. Lee D, Begley CE. Delays in Seeking Health Care: Comparison of Veterans and the General Population. *J Public Health Manag Pract*. Mar/Apr 2017;23(2):160-168. doi:10.1097/phh.0000000000000420

9. Fortney JC, Carey EP, Rajan S, Rise PJ, Gunzburger EC, Felker BL. A comparison of patient-reported mental health outcomes for the Department of Veterans Affairs' regional telehealth and Community Care Programs. *Health Serv Res*. Aug 2022;57(4):755-763. doi:10.1111/1475-6773.13993

10. Petros V, Tsambikos E, Madhoun M, Tierney WM. Impact of Community Referral on Colonoscopy Quality Metrics in a Veterans Affairs Medical Center. *Clin Transl Gastroenterol*. Jan 1 2022;13(3):e00460. doi:10.14309/ctg.0000000000000460

11. Heidenreich PA, Zapata A, Shieh L, Oliva N, Sahay A. Patient ratings of Veterans Affairs and affiliated hospitals. *Am J Manag Care*. Jun 2017;23(6):382-384.

12. Mody L, Greene MT, Saint S, et al. Comparing Catheter-Associated Urinary Tract Infection Prevention Programs Between Veterans Affairs Nursing Homes and Non-Veterans Affairs Nursing Homes. *Infect Control Hosp Epidemiol*. Mar 2017;38(3):287-293. doi:10.1017/ice.2016.279

13. Axon RN, Gebregziabher M, Everett CJ, Heidenreich P, Hunt KJ. Dual health care system use is associated with higher rates of hospitalization and hospital readmission among veterans with heart failure. *Am Heart J*. Apr 2016;174:157-63. doi:10.1016/j.ahj.2015.09.023

14. Shields MC, Rosenthal MB. Quality of Inpatient Psychiatric Care at VA, Other Government, Nonprofit, and For-Profit Hospitals: A Comparison. *Psychiatr Serv*. Mar 1 2017;68(3):225-230. doi:10.1176/appi.ps.201600074

15. Presley CJ, Kaur K, Han L, et al. Aggressive End-of-Life Care in the Veterans Health Administration versus Fee-for-Service Medicare among Patients with Advanced Lung Cancer. *J Palliat Med*. Jun 2022;25(6):932-939. doi:10.1089/jpm.2021.0436

16. Nuti SV, Qin L, Rumsfeld JS, et al. Association of Admission to Veterans Affairs Hospitals vs Non-Veterans Affairs Hospitals With Mortality and Readmission Rates Among Older Men Hospitalized With Acute Myocardial Infarction, Heart Failure, or Pneumonia. *JAMA*. Feb 9 2016;315(6):582-92. doi:10.1001/jama.2016.0278

17. Vanneman ME, Wagner TH, Shwartz M, et al. Veterans' Experiences With Outpatient Care: Comparing The Veterans Affairs System With Community-Based Care. *Health Aff (Millwood)*. Aug 2020;39(8):1368-1376. doi:10.1377/hlthaff.2019.01375

18. Gurewich D, Shwartz M, Beilstein-Wedel E, Davila H, Rosen AK. Did Access to Care Improve Since Passage of the Veterans Choice Act?: Differences Between Rural and Urban Veterans. *Med Care*. Jun 1 2021;59(Suppl 3):S270-S278. doi:10.1097/MLR.0000000000001490

19. Davila H, Rosen AK, Beilstein-Wedel E, Shwartz M, Chatelain L, Jr., Gurewich D. Rural Veterans' Experiences With Outpatient Care in the Veterans Health Administration Versus Community Care. *Med Care*. Jun 1 2021;59(Suppl 3):S286-S291. doi:10.1097/MLR.0000000000001552

20. Intrator O, O'Hanlon CE, Makineni R, Scott WJ, Saliba D. Comparing Post-Acute Populations and Care in Veterans Affairs and Community Nursing Homes. *J Am Med Dir Assoc*. Dec 2021;22(12):2425-2431.e7. doi:10.1016/j.jamda.2021.10.007

21. LaBedz SL, Krishnan JA, Chung YC, et al. Chronic Obstructive Pulmonary Disease Outcomes at Veterans Affairs Versus Non-Veterans Affairs Hospitals. *Chronic Obstr Pulm Dis*. Jul 28 2021;8(3):306-313. doi:10.15326/jcopdf.2021.0201

22. Gidwani R, Asch SM, Needleman J, et al. End-of-Life Cost Trajectories in Cancer Patients Treated by Medicare versus the Veterans Health Administration. *J Am Geriatr Soc*. Apr 2021;69(4):916-923. doi:10.1111/jgs.16941

23. Griffith KN, Ndugga NJ, Pizer SD. Appointment Wait Times for Specialty Care in Veterans Health Administration Facilities vs Community Medical Centers. *JAMA Netw Open*. Aug 3 2020;3(8):e2014313. doi:10.1001/jamanetworkopen.2020.14313

24. Gidwani-Marszowski R, Faricy-Anderson K, Asch SM, Illarmo S, Ananth L, Patel MI. Potentially avoidable hospitalizations after chemotherapy: Differences across medicare and the Veterans Health Administration. *Cancer*. Jul 15 2020;126(14):3297-3302. doi:10.1002/cncr.32896

25. Penn M, Bhatnagar S, Kuy S, et al. Comparison of Wait Times for New Patients Between the Private Sector and United States Department of Veterans Affairs Medical Centers. *JAMA Netw Open*. Jan 4 2019;2(1):e187096. doi:10.1001/jamanetworkopen.2018.7096

26. Makarov DV, Ciprut S, Walter D, et al. Association Between Guideline-Discordant Prostate Cancer Imaging Rates and Health Care Service Among Veterans and Medicare Recipients. *JAMA Netw Open*. Aug 3 2018;1(4):e181172. doi:10.1001/jamanetworkopen.2018.1172

27. Wang V, Coffman CJ, Stechuchak KM, et al. Survival among Veterans Obtaining Dialysis in VA and Non-VA Settings. *J Am Soc Nephrol*. Jan 2019;30(1):159-168. doi:10.1681/asn.2018050521

28. Thorpe CT, Gellad WF, Mor MK, et al. Effect of Dual Use of Veterans Affairs and Medicare Part D Drug Benefits on Antihypertensive Medication Supply in a National Cohort of Veterans with Dementia. *Health Serv Res*. Dec 2018;53 Suppl 3(Suppl Suppl 3):5375-5401. doi:10.1111/1475-6773.13055

29. Vercammen-Grandjean C, Schopfer DW, Zhang N, Whooley MA. Participation in Pulmonary Rehabilitation by Veterans Health Administration and Medicare Beneficiaries After Hospitalization for Chronic Obstructive Pulmonary Disease. *J Cardiopulm Rehabil Prev*. Nov 2018;38(6):406-410. doi:10.1097/hcr.0000000000000357

30. Wang V, Coffman CJ, Stechuchak KM, et al. Comparative Assessment of Utilization and Hospital Outcomes of Veterans Receiving VA and Non-VA Outpatient Dialysis. *Health Serv Res*. Dec 2018;53 Suppl 3(Suppl Suppl 3):5309-5330. doi:10.1111/1475-6773.13022

31. Anhang Price R, Sloss EM, Cefalu M, Farmer CM, Hussey PS. Comparing Quality of Care in Veterans Affairs and Non-Veterans Affairs Settings. *J Gen Intern Med*. Oct 2018;33(10):1631-1638. doi:10.1007/s11606-018-4433-7

32. Kurella Tamura M, Thomas IC, Montez-Rath ME, et al. Dialysis Initiation and Mortality Among Older Veterans With Kidney Failure Treated in Medicare vs the Department of Veterans Affairs. *JAMA Intern Med*. May 1 2018;178(5):657-664. doi:10.1001/jamainternmed.2018.0411

33. Barnett PG, Hong JS, Carey E, Grunwald GK, Joynt Maddox K, Maddox TM. Comparison of Accessibility, Cost, and Quality of Elective Coronary Revascularization Between Veterans Affairs and Community Care Hospitals. *JAMA Cardiol*. Feb 1 2018;3(2):133-141. doi:10.1001/jamacardio.2017.4843

34. Blay E, Jr., DeLancey JO, Hewitt DB, Chung JW, Bilimoria KY. Initial Public Reporting of Quality at Veterans Affairs vs Non-Veterans Affairs Hospitals. *JAMA Intern Med*. Jun 1 2017;177(6):882-885. doi:10.1001/jamainternmed.2017.0605

35. Jia H, Pei Q, Sullivan CT, et al. Poststroke Rehabilitation and Restorative Care Utilization: A Comparison Between VA Community Living Centers and VA-contracted Community Nursing Homes. *Med Care*. Mar 2016;54(3):235-42. doi:10.1097/mlr.0000000000000494

36. Watkins KE, Smith B, Akincigil A, et al. The Quality of Medication Treatment for Mental Disorders in the Department of Veterans Affairs and in Private-Sector Plans. *Psychiatr Serv*. Apr 1 2016;67(4):391-6. doi:10.1176/appi.ps.201400537

37. Chan DC, Danesh K, Costantini S, Card D, Taylor L, Studdert DM. Mortality among US veterans after emergency visits to Veterans Affairs and other hospitals: retrospective cohort study. *BMJ*. Feb 16 2022;376:e068099. doi:10.1136/bmj-2021-068099

38. Feyman Y, Asfaw DA, Griffith KN. Geographic Variation in Appointment Wait Times for US Military Veterans. *JAMA Netw Open*. Aug 1 2022;5(8):e2228783. doi:10.1001/jamanetworkopen.2022.28783

39. Pickering AN, Zhao X, Sileanu FE, et al. Assessment of Care Cascades Following Low-Value Prostate-Specific Antigen Testing Among Veterans Dually Enrolled in the US Veterans Health Administration and Medicare Systems. *JAMA Netw Open*. Dec 1 2022;5(12):e2247180. doi:10.1001/jamanetworkopen.2022.47180

40. Scheuner MT, Huynh AK, Chanfreau-Coffinier C, et al. Demographic Differences Among US Department of Veterans Affairs Patients Referred for Genetic Consultation to a Centralized VA Telehealth Program, VA Medical Centers, or the Community. *JAMA Netw Open*. Apr 1 2022;5(4):e226687. doi:10.1001/jamanetworkopen.2022.6687
